# Added Sugar, Sugar-Sweetened Beverages, and Artificially Sweetened Beverages and Risk of Cardiovascular Disease: Findings from the Women’s Health Initiative and a Network Meta-Analysis of Prospective Studies

**DOI:** 10.3390/nu14204226

**Published:** 2022-10-11

**Authors:** Bo Yang, Andrea J. Glenn, Qing Liu, Tracy Madsen, Matthew A. Allison, James M. Shikany, JoAnn E. Manson, Kei Hang Katie Chan, Wen-Chih Wu, Jie Li, Simin Liu, Kenneth Lo

**Affiliations:** 1 Global Health Research Center, Guangdong Provincial People’s Hospital, Guangdong Academy of Medical Sciences, Guangzhou 510030, China; 2 Centre for Global Cardiometabolic Health, Departments of Epidemiology and Medicine, Brown University, Providence, RI 02912, USA; 3 Department of Nutritional Sciences, University of Toronto, Toronto, ON M5S 1A8, Canada; 4 Toronto 3D Knowledge Synthesis and Clinical Trials Unit, Clinical Nutrition and Risk Factor Modification Centre, St. Michael’s Hospital, Toronto, ON M5C 2T2, Canada; 5 Department of Nutrition, Harvard T.H. Chan School of Public Health, Boston, MA 02115, USA; 6 Department of Emergency Medicine, Brown University, Providence, RI 02912, USA; 7 Department of Family Medicine, University of California San Diego, La Jolla, CA 92093, USA; 8 Division of Preventive Medicine, University of Alabama at Birmingham, Birmingham, AL 35294, USA; 9 Division of Preventive Medicine, Department of Medicine, Brigham and Women’s Hospital, Harvard Medical School, Boston, MA 02115, USA; 10 Department of Biomedical Sciences, Department of Electrical Engineering, City University of Hong Kong, Hong Kong, China; 11 Research Institute for Smart Ageing, The Hong Kong Polytechnic University, Hong Kong, China; 12 Department of Applied Biology and Chemical Technology, The Hong Kong Polytechnic University, Hung Hom, Hong Kong, China

**Keywords:** added sugar, sugar-sweetened beverages, artificially sweetened beverages, cardiovascular disease, network meta-analysis, prospective cohort

## Abstract

Much remains unknown about the role of added sugar in relation to cardiovascular disease (CVD) and the relative contributions of sugar-sweetened beverages (SSB) or artificially sweetened beverages (ASB) to CVD risk. Among the 109,034 women who participated in Women’s Health Initiative, we assessed average intakes of added sugar, SSB and ASB, and conducted Cox regression to estimate the hazard ratios (HRs) and their 95% confidence intervals for CVD risk. The consistency of findings was compared to a network meta-analysis of all available cohorts. During an average of 17.4 years of follow-up, 11,597 cases of total CVD (nonfatal myocardial infarction, coronary heart disease (CHD) death, stroke, coronary revascularization, and/or incident heart failure) were confirmed. Added sugar as % energy intake daily (%EAS) at ≥15.0% was positively associated with total CVD (HR = 1.08 [1.01, 1.15]) and CHD (HR = 1.20 [1.09, 1.32]). There was also a higher risk of total CVD associated with ≥1 serving of SSB intake per day (HR = 1.29 [1.17, 1.42]), CHD (1.35 [1.16, 1.57]), and total stroke (1.30 [1.10, 1.53]). Similarly, ASB intake was associated with an increased risk of CVD (1.14 [1.03, 1.26]) and stroke (1.24 [1.04, 1.48]). According to the network meta-analysis, there was a large amount of heterogeneity across studies, showing no consistent pattern implicating added sugar, ASB, or SSB in CVD outcomes. A diet containing %EAS ≥15.0% and consuming ≥1 serving of SSB or ASB may be associated with a higher CVD incidence. The relative contribution of added sugar, SSB, and ASB to CVD risk warrants further investigation.

## 1. Introduction

Added sugars are defined as sugars, sweeteners, and syrups that are consumed or used as ingredients in processed and prepared foods (e.g., ice cream, candy, and regular soft drinks) [1]. According to the data from the National Health and Nutrition Examination Survey (NHANES), sugar-sweetened beverages (SSB) are the most common items consumed by participants, with the highest deciles of added sugar intake across all age groups [2].

Few prospective studies have evaluated the direct relationship between added sugar and cardiovascular disease (CVD) risk [3]. Khan et al. reviewed 24 prospective cohort studies [3] and found only two that examined the role of added sugar in cardiovascular death [4,5], reporting a summary relative risk (95% confidence interval (CI)) of 1.03 [0.85, 1.26] for the relation between added sugar intake and cardiovascular mortality [3]. Subsequently, an updated systematic review [6] identified two additional studies implicating the role of higher % energy intake from added sugar (%EAS) in relation to CVD mortality [7,8]. In contrast, in a cohort of older adults in Hong Kong, Liu et al. reported an inverse association between added sugar intake and CVD mortality [9].

According to the American Heart Association, the influence of sugar substitutes on long-term cardiometabolic health remains controversial [10]. A recent meta-analysis reported that substituting artificially sweetened beverages (ASBs) for SSBs has benefits in terms of reducing body weight, body mass index (BMI), percentage of body fat, and intrahepatocellular lipid [11], whereas another pooled analysis of prospective cohorts indicated a positive association between ASB and CVD mortality, particularly when it is consumed at high intake levels (>2 servings per day) [12].

Moreover, fruit juices can be perceived as a healthier option to SSB [13]. Although there is also evidence linking fruit juice consumption with an increased risk of diabetes [14], its relation with CVD mortality remains uncertain [15], and few studies have attempted to comprehensively elucidate the associations of different sources of SSBs and ASBs with cardiometabolic health. Thus, the comparative influence of added sugar, SSBs, and ASBs on cardiometabolic health remains uncertain.

Due to the reduced level of ovarian hormones during menopause, postmenopausal women are at increased risk of CVD [16] and stroke [17], although few studies have investigated the effects of added sugar, SSBs, and ASBs in this population. To address this knowledge gap, we examined the association between the intake of added sugar, SSBs, ASBs, and the risk of CVD among postmenopausal women who participated in the Women’s Health Initiative (WHI) study and performed a network meta-analysis of available prospective studies.

## 2. Materials and Methods

### 2.1. Study Population and Design

**Figure 1:**
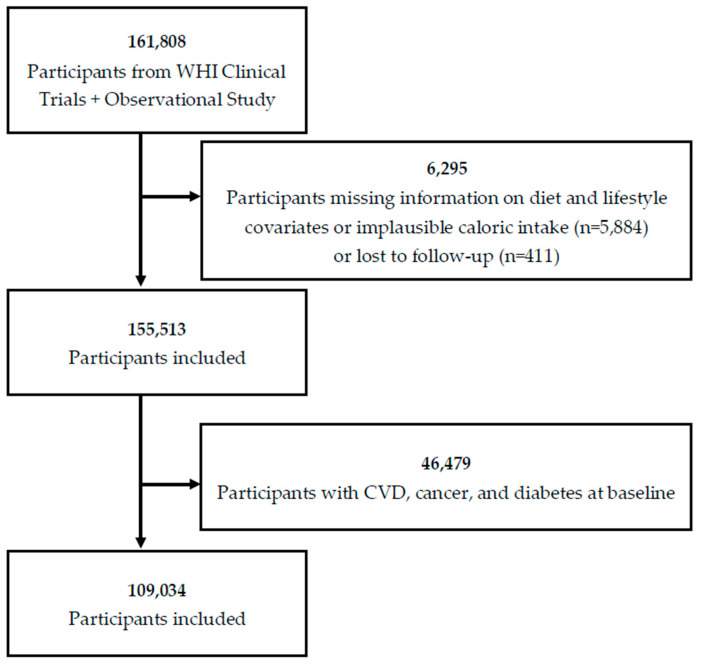
Flow chart for the study sample: Women’s Health Initiative (WHI) cohort, 1993–2021.

The design and methods of the WHI have been published elsewhere [18,19,20]. Between 1993 and 1998, postmenopausal women aged 50–79 years were recruited into a clinical trial or an observational study (OS) (*n* = 161,808). The current analysis includes follow-up through March 6, 2021. Participants who had missing information regarding diet and lifestyle covariates; implausible caloric intake (<600 kcal or >5000 kcal/day); were lost to follow-up; or had a history of CVD, cancer, or diabetes at baseline were excluded from the analysis, leaving 109,034 women in the final analytical cohort (Figure 1). Written informed consent was obtained from all participants, and the study protocol was approved by institutional review boards at all participating institutions.

### 2.2. Outcome Variables

Outcomes included total CVD, coronary heart disease (CHD; defined as incident fatal and nonfatal myocardial infarction (MI)), stroke, heart failure (HF), and total mortality. Total CVD is a composite of nonfatal myocardial infarction, CHD death, stroke, coronary revascularization, and incident HF [21]. Medical records and death certificates for all outcomes were reviewed by central physician adjudicators or trained local adjudicators [22]. Participants were followed from study enrollment to CVD onset, death, censuring, or end of study follow-up—whichever occurred first.

### 2.3. Exposure Variables

Added sugar and SSB intakes were assessed using a validated 122-item food frequency questionnaire (FFQ). The FFQ assessed the number of servings of food items rich in added sugar and computed the aggregate intake by using the nutrient database for the WHI FFQ adapted from the University of Minnesota Nutrition Coordinating Center (Minneapolis, MN, USA) Nutrient Database [23]. Added sugar was assessed as percent of energy from added sugars (%EAS [<10.0%, 10.0–14.9%, ≥15.0%]). The cut-off point was adapted from the World Health Organization (<10%EAS) [24], and we chose 15.0% as the highest cut-off value because it was approximately the highest quintile of intake within the WHI population (14.63%).

SSB consumption was calculated according to the average values from two FFQs at baseline and year 3 of assessment. SSB consumption was assessed in accordance with a previous WHI study that examined SSBs and diabetes incidence [25], wherein SSB consumption was defined as the sum of the consumption of regular soft drinks, fruit juices, and fruit drinks. In brief, participants were asked on the FFQ about how often they consumed 12-ounce glasses (335 mL) of regular soft drinks (not diet) and how often they drank 6-ounce glasses (177 mL) of “orange juice and grapefruit juice,” “other fruit juices such as apple and grape,” and “Tang, Kool-Aid, Hi-C, and other fruit drinks” during the past 3 months. Subsequently, a continuous variable was obtained for SSB consumption in terms of number of 12-ounce glasses (355 mL). We analyzed SSB consumption on the basis of three categories: <1 serving/week, 1 serving/week to <1 serving/day, and ≥1 servings/day, as described in a previous publication [26]. For the three components of SSBs (regular soft drinks, fruit juices, and fruit drinks), the categories were further divided into <1 serving/week and ≥1 serving/week. ASB consumption was assessed during year 3 of follow-up in OS participants only (*n* = 52,754). ASB consumption was assessed in terms of 12-ounce glasses (355 mL). Participants were asked how often they drank “diet drinks, such as Diet Coke or diet fruit drinks” in the past 3 months. The range of responses was the same as that of SSB.

### 2.4. Covariates

At baseline, participants completed the questionnaires on age and race/ethnicity, socioeconomic status (e.g., education in years), lifestyle factors (e.g., diet, smoking, alcohol use, and physical activity), and family history of diabetes or heart disease. Height and weight were measured at baseline to calculate the BMI, and systolic and diastolic blood pressures were measured.

### 2.5. Statistical Method for Cohort Analysis

Descriptive statistics were used to describe the frequencies, proportions, means, and standard deviations of the demographic characteristics. Descriptive statistics were shown according to intake levels. Significant differences in continuous and categorical variables between the groups were tested by one-way ANOVA and chi-square test, respectively, with a significance level of *p* < 0.05.

We used Cox proportional-hazard regression models to examine the association between %EAS, SSBs, and ASBs, as well as the CVD outcomes. Potential confounders were adjusted in the multivariable models [27,28,29]. Four multivariable models were used. Model 1 was adjusted for age (continuous), region (Northeast, South, Midwest, and West), smoking (never, past, and current), and study arm (hormone replacement therapy (HRT) arm, dietary modification (DM) arm, and calcium and vitamin D (CaD) arm). Model 2 was adjusted for model 1 + race/ethnicity (White, African American, Hispanic, and Asian), education (college or above and below college), marital status (presently married/other), BMI (continuous), physical activity (continuous), alcohol intake (>7 drinks/week, <7 drinks/week), energy intake (continuous), self-reported hypertension status (yes/no), family history of CVD (yes/no), family history of diabetes (yes/no), postmenopausal hormone use (never, past, and current), and cholesterol-lowering medication use (yes/no). Model 3 was adjusted for model 2 + total protein intake (grams/day), saturated fat intake (grams/day), trans fat intake (grams/day), and fiber intake (grams/day), excluding BMI. Model 4 was adjusted for model 3 + BMI. Schoenfeld residuals for fully adjusted models showed no evidence of violation of proportional hazard assumption.

We applied subgroup analyses according to BMI category (<25 kg/m2, 25 to <30 kg/m2, ≥30kg/m2) and physical activity (below and above the median level (8.75 metabolic equivalent of task (MET)-hours/wk)) to identify potential effect modifiers. To test the robustness of our findings, we conducted sensitivity analyses by (1) restricting the data to the OS participants only and (2) excluding incident cases of CVD events within the first 3 years of follow-up to address possible reverse causation. The statistical analyses were conducted with Stata statistical software (Stata Statistical Software: Release 17. College Station, TX, USA). Two-sided *p*-values less than 0.05 were considered statistically significant.

### 2.6. A Network Meta-Analysis of Prospective Cohorts

To evaluate the consistency and reliability of all available prospective data reported in the literature to date (1 April 2022), we also performed a systematic review and network meta-analysis including the present study and available cohorts that evaluated whether added sugar, SSBs, and ASBs are associated with CVD outcomes. The quality of included studies was assessed by the Newcastle–Ottawa scale (NOS). Each study was judged according to eight items from three categories: the selection of study groups, the comparability of cohorts on the basis of the design or analysis, and the outcome assessment. The maximum score per study is 9 points, with higher scores indicating better study quality. An NOS score of 7–9 can indicate good study quality according to the Agency for Healthcare Research and Quality (AHRQ) standards, 4–6 is fair, and 1–3 is poor.

Network meta-analysis is usually used to compare multiple interventions in a single analysis by combining both direct and indirect evidence across a network of studies [30]. In addition to interventions, prospective cohort studies can also be analyzed with this method, providing a more comprehensive understanding complementary to randomized controlled trials (RCTs) [30].

A random-effects network meta-analysis model with inverse variance weighting was fitted, and 95% CIs were determined in pooled estimates [31]. The fully adjusted effect estimates for the highest versus the lowest category of exposure were extracted from each included study. Heterogeneity across included studies was investigated via visual inspection, Cochrane’s Q statistic, and the inconsistency index (I^2^), with an I^2^ of at least 50% indicating moderate heterogeneity [31]. Pooled estimates and a network graph were plotted with the fitted network meta-analysis model, presenting the effect sizes of each dietary exposure and the overall structure of comparisons in the network. Finally, a ranking of added sugar, SSBs, and ASBs was generated, indicating which dietary exposure is more or less likely to cause the CVD outcomes. We also performed subgroup analysis by sex and leave-one-out analysis, i.e., omitting one study at a time and calculating a pooled estimate for the remainder of the studies to evaluate whether the results were affected markedly by a single study. Details of the literature search strategy are provided in the Supplementary Information (Table S1). The protocol was registered in PROSPERO (CRD42020200685).

## 3. Results

### 3.1. Demographic Features of Included Participants

Table 1 shows the characteristics of 109,034 postmenopausal women at baseline. Women in the highest %EAS (≥15.0%) group were younger, less active, and had higher BMI. Multiple demographic features also differed by %EAS. For example, individuals who were living in the northeast part of the United States, who were Black or African American, who had been treated for high cholesterol, who were current smokers, and who consumed ≥1 serving of SSB and ASB per day were more likely to have the highest level of %EAS (≥15.0%).

### 3.2. Associations between Added Sugar, SSBs, ASBs, and CVD Risk

After an average of 17.4 years of follow-up, 11,597 cases of cardiovascular disease events were confirmed. Compared with women with the lowest intake level (<1 serving/week), those consuming ≥1 serving/day of SSB had a higher risk of total CVD (1.29 [1.17, 1.42]), CHD (1.35 [1.16, 1.57]), and HF (1.35 [1.03, 1.76]) in the fully adjusted model (Table 2). For the subtypes of SSB, a higher risk of total CVD was observed among participants consuming ≥1 serving/week of fruit drinks (1.13 [1.03, 1.25]) and soft drinks (1.10 [1.04, 1.16]). In addition, consuming ≥1 serving/week of soft drinks was associated with a higher risk of CHD (1.17 [1.08, 1.27]), and consuming ≥1 serving/week of fruit drinks was associated with a higher risk of HF (1.61 [1.26, 2.06]). When using %EAS <10% as the referent, %EAS of 10–14.9% was not associated with risk of total CVD, but %EAS of ≥15.0% was associated with a higher risk of total CVD (1.08 [1.01, 1.15]) and CHD (1.20 [1.09, 1.32]) in the fully adjusted model (Model 3). In contrast, consuming ≥1 serving/week of fruit juice was associated with a lower risk of CHD (0.93 [0.87, 0.99]).

The association between added sugar, SSB, and the risk of stroke is presented in Table 3. A higher risk of stroke was observed among women consuming ≥ 1 serving/day of total SSBs (1.30 [1.10, 1.53)) and ≥1 serving/week of fruit drinks (1.19 [1.01, 1.39]). For ischemic stroke, a marginally lower risk was observed for women consuming 10–14.9%EAS (0.91 [0.83, 1.00]), with a significantly higher risk for those consuming ≥ 1 serving/day of total SSBs (1.32 [1.09, 1.59]). For hemorrhagic stroke, women consuming 10–14.9%EAS had a marginally higher risk (1.22 [1.00, 1.50]).

The association between ASB and the risk of all CVD outcomes is presented in Table 4. Consuming ≥ 1 serving/day of ASB was associated with an elevated risk of total CVD (1.14 [1.03, 1.26]) and total stroke (1.24 [1.04, 1.48]).

For the above regression models, the significance of association did not differ substantially with (model 4) or without (model 3) adjustment for BMI.

### 3.3. Subgroup and Sensitivity Analyses

We conducted subgroup analyses according to baseline BMI categories and levels of physical activity to identify potential effect modifiers (Table S2 through Table S5). The results remained largely consistent in each of the subgroup analyses, and we observed significant effect modification by BMI, i.e., the association of %EAS ≥ 15.0% with CHD (p for interaction = 0.01, Table S2) and that between consuming ≥ 1 serving/day of ASB with ischemic stroke (p for interaction = 0.02, Table S4). Both associations were significant only among women with BMI < 25 kg/m^2^ or those ≥ 30 kg/m^2^. The association of added sugar, SSB (Table S4), and ASB intake (Table S5) with CVD outcomes did not differ according to physical activity level.

We performed sensitivity analyses on the association between the intake of added sugar, SSBs, and ASBs with CVD outcomes (in the OS participants only, excluding CVD events within the first 3 years of follow-up) (Table S6 through Table S8) and found that the exposure–outcome associations were consistent across all analyses for added sugar and SSBs.

### 3.4. Results from the Network Meta-Analyses

We identified 21 cohort studies from the literature search that evaluated the relationship between added sugar [4,5,7,8,9], SSBs [32,33,34,35,36,37,38,39,40,41,42,43,44,45,46], ASBs [32,33,34,38,40,42,43,46], and CVD outcomes (CVD incidence, CVD mortality, stroke, CHD or MI) (Table S9, Figure S1). All included studies were of good quality, with NOS scores of 7–9, except one of fair quality, with an NOS score of 6 (Table S10) [44]. Two studies that investigated the association between ASB and CVD used the same data sources as the present study but with different selection criteria [26,47]. Therefore, we narratively compared our findings with the results of these studies in the Discussion section. Studies that analyzed the association between SSBs and CVD outcomes were the most frequent combination, and studies that examined the association between added sugar and CVD outcomes were the least frequent in the literature (Figure S2).

**Figure 2:**
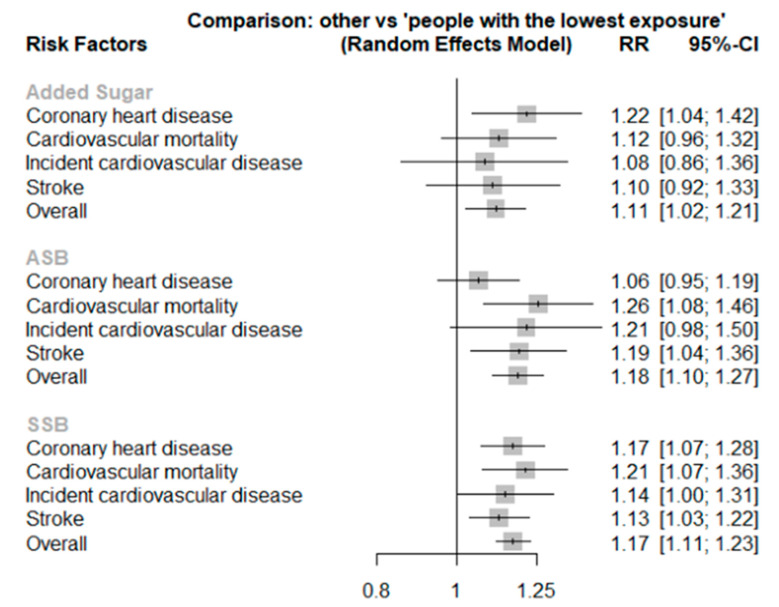
Random effects network meta-analyses on added sugar, SSB, ASB, and the risk of cardiovascular outcomes. The network meta-analysis was performed using frequentist methods with a random effects model. The study effect sizes for the highest versus the lowest category of exposure were calculated. Abbreviations: ASB, artificially sweetened beverages; RR, relative risk; SSB, sugar-sweetened beverages.

The intake of added sugar was significantly associated with CHD (1.22 [1.04, 1.42]) but not other outcomes (Table 5, Figure 2). The highest category of ASBs was associated with cardiovascular mortality (1.26 [1.08, 1.46]), in addition to stroke (1.19 [1.04, 1.36]). SSB intake was also directly related to an increased risk of CVD (1.14 [1.00, 1.31]), cardiovascular mortality (1.21 [1.07, 1.36]), CHD (1.17 [1.07, 1.28]), and total stroke (1.13 [1.03, 1.22]). Cardiovascular mortality and stroke shared the same rankings of exposures, where ASBs had the highest ranking, followed by SSBs and added sugar.

The relationship between SSB intake and cardiovascular mortality and CHD appeared more evident in women (1.34 [1.01, 1.77]) than in men (1.17 [0.99, 1.38]). In addition, the SSB–stroke relation was stronger in women than in men (women 1.22 [1.12, 1.32], men 1.02 [0.83, 1.25]) (Table S11). The magnitude of association between added sugar, SSB, ASB intake, and CVD outcomes was consistent when excluding one study at a time in leave-one-out analysis (Table S12).

## 4. Discussion

### 4.1. Interpretation of Main Findings

The present analysis involved postmenopausal women in the WHI study and revealed that a %EAS of ≥15.0% was associated with an increased risk of total CVD and CHD. The consumption of ≥1 serving of SSB per day was associated with a modestly elevated risk of total CVD, CHD, and total stroke. ASB consumption was also associated with an increased risk of total CVD and total stroke.

Compared to previous studies that mainly focused on CVD mortality [3,6], our work has further explored the role of added sugar with respect to the risk of multiple CVD outcomes, along with the association of SSBs and ASBs with CVD risk. Although the present study has strengthened the evidence on the association between added sugar and CVD risk, there are several major challenges to be overcome in terms of research methods. The first challenge is to identify accurate and objective biomarkers for added sugar [48]. Compared with glycemic indicators (e.g., glycemic load) that have been validated by postprandial glucose response [49], using biomarkers for validation is not as successful for added sugar due to the chemical indistinguishability between naturally occurring and added forms of the same sugar molecule [50]. Because SSBs are the most common source of added sugar intake in the U.S. [2], it is unclear whether the significant associations are solely contributed by added sugar or due to SSBs.

Another issue to be resolved is whether the sources of added sugar have differing impacts on CVD. For example, the Mr. Osteoporosis & Ms. Osteoporosis Study and NIH-AARP Diet Health Study examined the source-specific association between added sugar and CVD mortality [5,9] instead of treating added sugar intake as an aggregate exposure or performing analysis on sugar-rich food or beverages [4,7]. No significant associations between sources of added sugar and CVD were found in the Mr. Osteoporosis & Ms. Osteoporosis Study [9]. In the NIH-AARP Diet Health Study, the highest intake of added sugar from beverages was associated with a higher risk for CVD death among women (1.13 [1.01, 1.26]) but not men (1.01 [0.94, 1.09]). Added sugar from solid foods was associated with lower risk of CVD death among men (0.78 [0.72, 0.85]) and women (0.81 [0.73, 0.91]) [5]. Furthermore, consuming ≥ 1 serving/week of fruit juice was associated with a lower risk of CHD (0.93 [0.87, 0.99]), although we were not able to quantify the amount of added sugar in the fruit juice. The discrepancy in findings with respect to added sugar according to source has highlighted the limitation of assessing added sugar at an aggregate level.

In the WHI cohort, the association between SSBs and total CVD (1.29, [1.17, 1.42]) agreed with the results of the current network meta-analysis (1.14 [1.00, 1.31]). The association between SSB and CHD in the present study (1.35 [1.16, 1.57]) was also consistent with our pooled result (1.17 [1.07, 1.28]). In our study, we further analyzed the association between individual SSBs and CVD risk; the consumption of ≥1 serving of soft drinks per week was associated with a 17% higher risk of CVD mortality in the primary analysis. Moreover, at least one serving of ASB intake per day was significantly associated with total CVD (1.14 [1.03, 1.26]). The present study and the two previous publications from the WHI examined the association between ASB intake and CVD risk in a similar group of participants [26,47], although in the present study, we excluded participants with pre-existing CVD, diabetes, and cancer at baseline to minimize the issue of reverse causation. Interestingly, another recent meta-analysis of cohort studies used change analyses of repeated measures of intake and substitution analyses to investigate the association of low- and no-calorie sweetened beverages (LNCSBs) consumption with cardiometabolic outcomes, revealing that substitution of LNCSBs for SSBs was associated with lower body weight, lower obesity and CHD incidence, and lower risk of CVD mortality [51]. The authors postulated that participants with higher LNCSB intake usually had a higher cardiometabolic risk, which is common in prospective cohort studies that rely largely on baseline or prevalent intakes of LNCSB [51].

There has been increasing evidence for the plausible biological mechanisms of the associations between added sugar and CVD risk. There will be a rapid increase in serum glucose and insulin concentrations after SSBs consumption [52]. This induces a high glycemic load, which leads to excessive weight gain, inflammation, and insulin resistance [53]. These adverse effects can cause metabolic syndrome, which is a major risk factor for CVD [54]. Excessive SSB consumption was independently associated with increased blood pressure in 2696 middle-aged men and women in U.S. and United Kingdom, which also suggests that SSBs may increase CVD risk by increasing blood pressure [55]. Moreover, excessive sugar intake was also associated with increased liver lipogenesis, hepatic triglyceride synthesis, and triglyceride levels, all of which increase the risk of CVD [56]. The mechanisms behind the association of ASB consumption and higher total CVD with total stroke risk are likely different from that of SSBs, as ASBs contain few to no calories. Potential mechanisms include forming a habit toward overeating sweets [57], possible alteration of gut microbiota composition [58], and increased levels of proinflammatory advanced glycation end products, contributing to the caramel coloring in ASBs [59].

With the use of network meta-analysis, we examined the mutual ranking of added sugar, ASBs, and SSBs based on their associations with cardiovascular outcomes. Although the interstudy heterogeneity was high, ASBs had the highest ranking, followed by SSBs and added sugar for cardiovascular mortality and stroke but not for other CVD outcomes. One possible reason for the heterogeneity in findings is the differential misclassifications of exposures. The effect estimates for the highest versus the lowest category of exposure were extracted from each included study, but the categorization of highest and lowest categories varied in each study. Another possible reason is the limited number of studies investigating some outcomes, as only four studies assessed incident CVD. Future research should consider less-studied outcomes to provide a more comprehensive view on added sugar, SSBs, ASBs, and different CVD outcomes. Furthermore, studies including direct comparisons between SSBs and ASBs are also needed to provide additional information on their role in cardiometabolic risk. To increase the comparability of findings, studies should adapt consistent categorizations in terms of exposure intake.

In our study, we addressed the ongoing controversy of how added sugar may relate to CVD outcomes and compared the results with SSB and ASB intake within the WHI population. Although the source-specific association needs to be verified in other cohorts, our analysis supports limiting added sugar to less than 15%EAS of the whole diet. Our study also confirmed the positive association of SSB and ASB intake with CVD outcomes. We strongly encourage future epidemiological studies to assess the effect of changes in ASB intake in minimizing the issue of possible reverse causation.

### 4.2. Strengths and Limitations

This study has several strengths. The current analysis included data from a large cohort with long-term follow-up and high-quality outcome assessment. We also performed network meta-analysis to evaluate the relative contribution of each dietary exposure on CVD outcomes. However, this study is also subject to several limitations. First, the present cohort included only postmenopausal women; therefore, we were not able to explore sex-specific associations. Second, we did not analyze source-specific associations between added sugar and CVD outcomes because added sugar intake was assessed as an aggregate exposure beyond SSBs. Third, dietary assessment was conducted at baseline and year 3 of follow-up only; therefore, the influence of dietary changes throughout the cohort on long-term disease risk is not clear. The relationship between diet and CVD outcomes might be weakened over time. Last but not least, there might be residual confounding factors, such as the changes in medication and lifestyle factors throughout the cohort. Despite the above limitations, this study contributes important dimensions to our understanding of how added sugar, SSBs, and ASBs might affect CVD risk.

## 5. Conclusions

In the present study of postmenopausal women in the United States, %EAS of ≥15.0% and consuming ≥1 serving of SSB or ASB per day were associated with a modestly increased risk of total CVD. As demonstrated by our network meta-analysis, the relative contribution of added sugar, SSBs, and ASBs to CVD risk still warrants further investigation.

## Figures and Tables

**Table 1:** Baseline characteristics of 109,034 participants initially free of cardiovascular disease in the Women’s Health Initiative According to percent energy from added sugars.

	% Energy from Added Sugar (%EAS)			
Mean (SD)/No. (%)	<10.0% (N = 48,537)	10.0–14.9% (N = 39,857)	≥15.0% (N = 20,640)	*p*-Value
Time to event/censored in years	17.5 (7.1)	17.6 (7.2)	16.9 (7.4)	<0.001
Age, years	62.8 (7.0)	62.6 (7.1)	61.6 (7.2)	<0.001
Body mass index, kg/m^2^	26.4 (5.6)	27.6 (5.7)	28.2 (6.0)	<0.001
Physical activity, MET hours per week	13.8 (14.3)	12.9 (13.8)	11.1 (13.5)	<0.001
Dietary Energy, kcal/day	1628 (619)	1672 (634)	1629 (691)	<0.001
Region in U.S.				<0.001
Northeast	10,237 (21)	9946 (25)	5285 (26)	
South	11,266 (23)	9912 (25)	6239 (30)	
Midwest	10,836 (22)	8863 (22)	4254 (21)	
West	16,198 (33)	11,134 (28)	4863 (24)	
Self-identified race and ethnicity				<0.001
White	41,607 (86)	34,215 (86)	15,986 (78)	
Black or African American	2665 (5)	2810 (7)	3037 (15)	
Hispanic	2015 (4)	1364 (4)	941 (5)	
Asian	1633 (3)	950 (2)	398 (2)	
Marital status, present relationship	32,234 (67)	25,672 (65)	12,051 (59)	<0.001
7+ alcoholic drinks per week	9286 (19)	3414 (9)	971 (5)	<0.001
Hormone therapy use				<0.001
Never used	14,799 (32)	12,560 (33)	7116 (35)	
Past user	9764 (21)	8272 (21)	4594 (23)	
Current user	22,363 (48)	17,753 (46)	8344 (42)	
Treated high cholesterol	4686 (10)	4193 (11)	2356 (12)	<0.001
History of hypertension	13,763 (29)	10,980 (28)	5927 (29)	0.004
Family history of diabetes	14,373 (30)	12,092 (31)	6299 (31)	<0.001
Family history of CVD	31,311 (65)	25,910 (65)	13,158 (64)	0.006
Smoking status				<0.001
Nevers	3991 (50)	21,039 (53)	10,490 (52)	
Past smoker	20,734 (43)	16,117 (41)	7967 (39)	
Current smoker	3199 (7)	2240 (6)	1906 (9)	
College education or above	34,347 (71)	27,383 (69)	12,886 (63)	<0.001
HT arm				<0.001
Not randomized to HT	40,024 (83)	32,785 (83)	16,249 (79)	
E alone	1520 (3)	1116 (3)	855 (4)	
E alone control	1460 (3)	1310 (3)	866 (4)	
E + P intervention	2820 (6)	2355 (6)	1375 (7)	
E + P control	2713 (6)	2241 (6)	1295 (6)	
DM arm				<0.001
Not randomized to DM	32,270 (66)	26,556 (67)	14,198 (69)	
Intervention	6489 (13)	5333 (13)	2586 (12)	
Control	9778 (20)	7968 (20)	3856 (19)	
CaD arm				0.789
Not randomized to CaD	36,445 (75)	29,943 (75)	15,446 (75)	
Intervention	6026 (12)	4989 (12)	2633 (13)	
Control	6066 (12)	4925 (12)	2561 (12)	
SSB consumption in servings				<0.001
<1/week	19,840 (41)	13,464 (34)	4681 (23)	
1/week to <1/day	27,603 (57)	24,548 (62)	11,806 (57)	
≥1 serving/day	1094 (2)	1845 (5)	4153 (20)	
ASB consumption in servings ^1^				<0.001
<1/week	16,102 (68)	12,212 (62)	5917 (63)	
1/week to <1/day	5172 (22)	4866 (25)	2010 (21)	
≥1 serving/day	2586 (11)	2390 (12)	1499 (16)	
Fruit juice in servings				<0.001
<1 serving/week	22,659 (47)	18,689 (47)	10,911 (53)	
≥1 serving/week	25,878 (53)	21,168 (53)	9729 (47)	
Fruit drinks in servings				<0.001
<1 serving/week	47,801 (98)	37,971 (95)	18,354 (89)	
≥1 serving/week	736 (2)	1886 (5)	2286 (11)	
Soft drinks in servings				<0.001
<1 serving/week	45,549 (94)	31,178 (78)	9920 (48)	
≥1 serving/week	2998 (6)	8679 (22)	10,720 (52)	

Abbreviations: CaD, calcium and vitamin D; DM, dietary modification; E alone, estrogen alone; E + P, estrogen plus progestin; HT, hormone therapy; Kcal, kilocalories; MET, metabolic equivalents; Q, quartile; SD, standard deviation; U.S., United States.^1^ ASB consumption was assessed during year 3 of follow-up and in the observational study (OS) participants only.

**Table 2:** Prospective association of added sugars and SSBs with total cardiovascular disease, coronary heart disease, and heart failure among 109,034 participants in the Women’s Health Initiative (CT + OS) (1993–2021).

			Model 1 *		Model 2 **		Model 3 ***		Model 4 ****	
	Cases/ Total	Person-Years	HR (95% CI)	*p*-Value	HR (95% CI)	*p* Value	HR (95% CI)	*p*-Value	HR (95% CI)	*p* Value
***TOTAL CARDIOVASCULAR DISEASE***										
%EAS										
<10%	5057/ 48,537	816,087	1.00 reference		1.00 reference		1.00 reference		1.00 reference	
10-14.9%	4209/ 39,857	669,565	1.03 (0.99, 1.07)	0.18	1.01 (0.97, 1.06)	0.65	1.00 (0.96, 1.06)	0.74	1.01 (0.97, 1.06)	0.62
≥15.0%	2331/ 20,640	331,972	1.18 (1.12, 1.24)	<0.001	1.08 (1.02, 1.14)	0.01	1.08 (1.01, 1.15)	0.02	1.08 (1.01, 1.15)	0.02
Total SSBs										
<1/week	4089/ 37,985	635,576	1.00 reference		1.00 reference		1.00 reference		1.00 reference	
1/week to <1 day	6713/ 63,957	1,068,255	1.02 (0.98, 1.07)	0.25	1.00 (0.96, 1.05)	0.99	0.99 (0.95, 1.04)	0.85	1.00 (0.96, 1.05)	0.91
≥ 1/day	795/ 7092	113,793	1.45 (1.34, 1.56)	<0.001	1.27 (1.17, 1.36)	<0.001	1.30 (1.18, 1.42)	<0.001	1.29 (1.17, 1.42)	<0.001
Fruit Juices										
<1/week	5669/ 52,259	864,018	1.00 reference		1.00 reference		1.00 reference		1.00 reference	
≥1/week	5928/ 56,775	953,607	0.98 (0.95, 1.02)	0.37	0.99 (0.94, 1.03)	0.50	0.97 (0.94, 1.02)	0.33	0.99 (0.95, 1.03)	0.56
Fruit drinks										
<1/week	11,020/ 104,126	1,742,044	1.00 reference		1.00 reference		1.00 reference		1.00 reference	
≥1/week	577/ 4908	75,581	1.30 (1.19, 1.41)	<0.001	1.14 (1.03, 1.25)	0.01	1.14 (1.03, 1.25)	<0.001	1.13 (1.03, 1.25)	0.01
Soft drinks										
<1/week	9126/ 86,647	1,460,115	1.00 reference		1.00 reference		1.00 reference		1.00 reference	
≥1/week	2471/ 22,387	357,510	1.24 (1.18, 1.30)	<0.001	1.11 (1.06, 1.17)	<0.001	1.10 (1.05, 1.17)	<0.001	1.10 (1.04, 1.16)	<0.001
***CORONARY HEART DISEASE***										
%EAS										
<10%	2055/ 48,537	840,422	1.00 reference		1.00 reference		1.00 reference		1.00 reference	
10-14.9%	1657/ 39,857	689,954	1.00 (0.94, 1.07)	0.96	0.99 (0.23, 1.07)	0.83	1.01 (0.94, 1.09)	0.77	1.02 (0.94, 1.09)	0.69
≥15.0%	983/ 20,640	342,816	1.22 (1.13, 1.32)	<0.001	1.14 (1.04, 1.24)	<0.01	1.19 (1.08, 1.31)	<0.001	1.20 (1.09, 1.32)	<0.001
Total SSBs										
<1/week	1703/ 37,985	654,137	1.00 reference		1.00 reference		1.00 reference		1.00 reference	
1/week to <1 day	2670/ 63,957	1,101,210	0.99 (0.93, 1.05)	0.72	0.96 (0.90, 1.03)	0.27	0.97 (0.90, 1.04)	0.42	0.98 (0.91, 1.05)	0.52
≥ 1/day	322/ 7092	117,846	1.44 (1.27, 1.63)	<0.001	1.26 (1.10, 1.45)	<0.001	1.34 (1.16, 1.56)	<0.001	1.35 (1.16, 1.57)	<0.001
Fruit Juices										
<1/week	2369/ 52,259	889,998	1.00 reference		1.00 reference		1.00 reference		1.00 reference	
≥1/week	2326/ 56,775	983,195	0.93 (0.88, 0.99)	0.02	0.92 (0.86, 0.98)	0.01	0.92 (0.86, 0.99)	0.02	0.93 (0.87, 0.99)	0.03
Fruit drinks										
<1/week	4472/ 104,126	1,794,920	1.00 reference		1.00 reference		1.00 reference		1.00 reference	
≥1/week	223/ 4908	78,273	1.24 (1.08, 1.42)	<0.001	0.98 (0.84, 1.15)	0.85	1.00 (0.86, 1.17)	0.98	0.99 (0.85, 1.17)	0.94
Soft drinks										
<1/week	3683/ 86,647	1,503,955	1.00 reference		1.00 reference		1.00 reference		1.00 reference	
≥1/week	1012/ 22,387	369,238	1.27 (1.18, 1.37)	<0.001	1.16 (1.07, 1.26)	<0.001	1.17 (1.08, 1.28)	<0.001	1.17 (1.08, 1.27)	<0.001
***HEART FAILURE***										
%EAS										
<10%	646/ 48,537	848,296	1.00 reference		1.00 reference		1.00 reference		1.00 reference	
10-14.9%	496/ 39,857	696,141	0.96 (0.86, 1.09)	0.54	0.96 (0.84, 1.10)	0.57	0.94 (0.82 1.08)	0.37	0.96 (0.84, 1.10)	0.56
≥15.0%	279/ 20,640	364,555	1.10 (0.95, 1.27)	0.19	1.02 (0.87, 1.20)	0.81	0.99 (0.83, 1.19)	0.95	1.01 (0.84, 1.20)	0.94
Total SSBs										
<1/week	469/ 37,985	661,580	1.00 reference		1.00 reference		1.00 reference		1.00 reference	
1/week to <1 day	853/ 63,957	1,110,471	1.07 (0.95, 1.20)	0.24	1.03 (0.90, 1.17)	0.70	1.01 (0.88, 1.15)	0.91	1.03 (0.90, 1.18)	0.64
≥ 1/day	99/ 7092	118,942	1.51 (1.21, 1.89)	<0.001	1.32 (1.03 1.69)	0.03	1.33 (1.02, 1.73)	0.03	1.35 (1.03, 1.76)	0.03
Fruit Juices										
<1/week	644/ 52,259	899,899	1.00 reference		1.00 reference		1.00 reference		1.00 reference	
≥1/week	777/ 56,775	991,093	1.06 (0.95, 1.18)	0.29	1.07 (0.95, 1.21)	0.26	1.05 (0.93, 1.19)	0.44	1.08 (0.96, 1.22)	0.22
Fruit drinks										
<1/week	1336/ 104,126	1,812,274	1.00 reference		1.00 reference		1.00 reference		1.00 reference	
≥1/week	85/ 4908	78,720	1.61 (1.29, 2.01)	<0.001	1.61 (1.29, 2.05)	<0.001	1.64 (1.28, 2.09)	<0.001	1.61 (1.26, 2.06)	<0.001
Soft drinks										
<1/week	1108/ 86,647	1,518,384	1.00 reference		1.00 reference		1.00 reference		1.00 reference	
≥1/week	313/ 22,387	372,609	1.32 (1.16, 1.50)	<0.001	1.09 (0.94, 1.27)	0.26	1.09 (0.94, 1.28)	0.26	1.07 (0.92, 1.26)	0.37

Abbreviations: CT = clinical trial; %EAS = percent energy from added sugar; HR = hazard ratio; OS = observational study; SSB = sugar-sweetened beverage. Under/over energy reporters and those with baseline CVD, diabetes, and cancer were excluded from the analysis. Total CVD is a composite of incidence and death from CHD, stroke, heart failure, and coronary revascularization (CABG or PTCA). * Model 1 adjusted for age, region, smoking, and study arm. ** Model 2 adjusted for model 1+ ethnicity, education, marital status, BMI, physical activity, alcohol intake, energy intake, hypertension status, family history of CVD, family history of diabetes, hormone therapy use, and cholesterol-lowering medication use. *** Model 3 adjusted for model 2 + total protein intake, saturated fat intake, trans fat intake, and fiber intake, excluding BMI. **** Model 4 adjusted for model 3 with BMI.

**Table 3:** Prospective association of added sugars and SSBs with stroke and stroke subtypes among 109,034 participants in the Women’s Health Initiative (CT + OS) (1993–2021).

			Model 1 *		Model 2 **		Model 3 ***		Model 4 ****	
	Cases/ Total	Person Years	HR (95% CI)	*p* Value	HR (95% CI)	*p* Value	HR (95% CI)	*p* Value	HR (95% CI)	*p* Value
***STROKE***										
%EAS										
<10%	1876/ 48,537	842,491	1.00 reference		1.00 reference		1.00 reference		1.00 reference	
10-14.9%	1518/ 39,857	691,427	1.00 (0.93, 1.07)	0.98	0.98 (0.91, 1.06)	0.59	0.97 (0.89, 1.05)	0.40	0.97 (0.90, 1.05)	0.49
≥15.0%	821/ 20,640	344,125	1.13 (1.04, 1.22)	<0.01	1.01 (0.92, 1.11)	0.83	0.99 (0.89, 1.10)	0.84	0.99 (0.89, 1.10)	0.83
Total SSBs										
<1/week	1469/ 37,985	657,070	1.00 reference		1.00 reference		1.00 reference		1.00 reference	
1/week to <1 day	2459/ 63,957	110,294	1.07 (1.00, 1.14)	0.06	1.02 (0.95, 1.10)	0.63	1.02 (0.94, 1.10)	0.64	1.02 (0.95, 1.10)	0.61
≥ 1/day	287/ 7092	118,026	1.54 (1.36, 1.76)	<0.001	1.29 (1.11, 1.50)	<0.01	1.32 (1.13, 1.56)	0.001	1.30 (1.10, 1.53)	<0.01
Fruit Juices										
<1/week	2025/ 52,259	893,601	1.00 reference		1.00 reference		1.00 reference		1.00 reference	
≥1/week	2190/ 56,775	984,442	1.04 (0.98, 1.11)	0.20	1.03 (0.96, 1.10)	0.44	1.03 (0.96, 1.10)	0.45	1.03 (0.96, 1.10)	0.44
Fruit drinks										
<1/week	3995/ 104,126	1,799,783	1.00 reference		1.00 reference		1.00 reference		1.00 reference	
≥1/week	220/ 4908	78,280	1.37 (1.20, 1.57)	<0.001	1.19 (1.02, 1.39)	0.03	1.19 (1.02, 1.39)	0.03	1.19 (1.01, 1.39)	0.03
Soft drinks										
<1/week	3340/ 86,647	1,508,172	1.00 reference		1.00 reference		1.00 reference		1.00 reference	
≥1/week	875/ 22,387	369,871	1.22 (1.13, 1.31)	<0.001	1.08 (0.99, 1.18)	0.09	1.06 (0.98, 1.16)	0.20	1.06 (0.97, 1.17)	0.20
***ISCHEMIC STROKE***										
%EAS										
<10%	1413/ 48,537	843,547	1.00 reference		1.00 reference		1.00 reference		1.00 reference	
10-14.9%	1092/ 39,857	692,535	0.95 (0.88, 1.03)	0.25	0.92 (0.84, 1.01)	0.07	0.90 (0.83, 0.99)	0.03	0.91 (0.83, 1.00)	0.04
≥15.0%	609/ 20,640	344,799	1.11 (1.01, 1.23)	0.03	0.98 (0.88, 1.09)	0.70	0.94 (0.85, 1.06)	0.36	0.94 (0.83, 1.06)	0.34
Total SSBs										
<1/week	1073/ 37,985	658,074	1.00 reference		1.00 reference		1.00 reference		1.00 reference	
1/week to <1 day	1820/ 63,957	110,457	1.09 (1.01, 1.17)	0.04	1.03 (0.94, 1.12)	0.53	1.02 (0.94, 1.12)	0.72	1.03 (0.94, 1.13)	0.51
≥ 1/day	221/ 7092	118,236	1.63 (1.41, 1.89)	<0.001	1.31 (1.10, 1.56)	<0.01	1.35 (1.11, 1.62)	0.002	1.32 (1.09, 1.59)	<0.01
Fruit Juices										
<1/week	1493/ 52,259	894,973	1.00 reference		1.00 reference		1.00 reference		1.00 reference	
≥1/week	1621/ 56,775	985,907	1.05 (0.98, 1.13)	0.16	1.04 (0.96, 1.13)	0.35	1.10 (0.96, 1.13)	0.36	1.04 (0.96, 1.13)	0.34
Fruit drinks										
<1/week	2951/ 104,126	1,802,449	1.00 reference		1.00 reference		1.00 reference		1.00 reference	
≥1/week	163/ 4908	78,432	1.38 (1.17, 1.61)	<0.001	1.19 (1.00, 1.43)	0.05	1.19 (0.99, 1.43)	0.06	1.19 (0.99, 1.42)	0.07
Soft drinks										
<1/week	2452/ 86,647	1,510,389	1.00 reference		1.00 reference		1.00 reference		1.00 reference	
≥1/week	662/ 22,387	370,492	1.25 (1.14, 1.36)	<0.001	1.07 (0.97, 1.19)	0.19	1.06 (0.95, 1.18)	0.31	1.05 (0.94, 1.17)	0.37
***HEMORRHAGIC STROKE***										
%EAS										
<10%	268/ 48,537	851,915	1.00 reference		1.00 reference		1.00 reference		1.00 reference	
10-14.9%	253/ 39,640	698,695	1.16 (0.97, 1.38)	0.09	1.21 (0.99, 1.47)	0.06	1.20 (0.98, 1.46)	0.08	1.22 (1.00, 1.50)	0.05
≥15.0%	129/ 20,640	348,013	1.18 (0.96, 1.46)	0.12	1.20 (0.94, 1.53)	0.14	1.22 (0.94, 1.59)	0.14	1.23 (0.94, 1.61)	0.13
Total SSBs										
<1/week	230/ 37,985	663,865	1.00 reference		1.00 reference		1.00 reference		1.00 reference	
1/week to <1 day	377/ 63,957	1,115,239	1.01 (0.85, 1.19)	0.93	1.00 (0.83, 1.22)	0.96	0.99 (0.82, 1.21)	0.94	1.00 (0.82, 1.22)	0.97
≥ 1/day	43/ 7092	119,519	1.29 (0.93, 1.80)	0.13	1.37 (0.94, 1.98)	0.10	1.35 (0.91, 2.00)	0.14	1.37 (0.92, 2.04)	0.13
Fruit Juices										
<1/week	308/ 52,259	903,166	1.00 reference		1.00 reference		1.00 reference		1.00 reference	
≥1/week	342/ 56,775	995,457	1.04 (0.89, 1.22)	0.64	1.00 (0.84, 1.20)	0.99	1.08 (1.07, 1.09)	0.90	0.99 (0.82, 1.19)	0.92
Fruit drinks										
<1/week	613/ 104,126	1,819,355	1.00 reference		1.00 reference		1.00 reference		1.00 reference	
≥1/week	37/ 4908	79,268	1.44 (1.03, 2.01)	0.03	1.41 (0.97, 2.05)	0.07	1.37 (0.94, 2.01)	0.09	1.39 (0.95, 2.04)	0.09
Soft drinks										
<1/week	523/ 86,647	1,524,368	1.00 reference		1.00 reference		1.00 reference		1.00 reference	
≥1/week	127/ 22,387	374,255	1.08 (0.89, 1.31)	0.45	1.12 (0.89, 1.40)	0.34	1.09 (0.86, 1.37)	0.49	1.11 (0.88, 1.41)	0.37

Abbreviations: CT = clinical trial; %EAS = percent energy from added sugar; HR = hazard ratio; OS = observational study; SSB = sugar-sweetened beverage. Under/over energy reporters and those with baseline CVD, diabetes, and cancer were excluded from the analysis. * Model 1 adjusted for age, region, smoking, and study arm. ** Model 2 adjusted for model 1+ ethnicity, education, marital status, BMI, physical activity, alcohol intake, energy intake, hypertension status, family history of CVD, family history of diabetes, hormone therapy use, and cholesterol-lowering medication use. *** Model 3 adjusted for model 2 + total protein intake, saturated fat intake, trans fat intake, and fiber intake, excluding BMI. **** Model 4 adjusted for model 3 with BMI.

**Table 4:** Prospective association of ASB servings with risk of cardiovascular disease outcomes among 52,754 participants in the Women’s Health Initiative (OS) (1993–2021).

			Model 1 *		Model 2 **		Model 3 ***		Model 4 ****	
	Cases/ Total	Person Years	HR (95% CI)	*p* Value	HR (95% CI)	*p* Value	HR (95% CI)	*p* Value	HR (95% CI)	*p* Value
***TOTAL CARDIOVASCULAR DISEASE***										
<1/week	2994/ 34,231	579,025	1.00 reference		1.00 reference		1.00 reference		1.00 reference	
1/week to <1 day	971/ 12,048	206,631	1.06 (0.98, 1.14)	0.14	0.97 (0.89, 1.05)	0.41	0.98 (0.92, 1.08)	0.95	0.97 (0.90, 1.05)	0.44
≥ 1 serving/day	536/ 6475	112,782	1.26 (1.15, 1.39)	<0.001	1.15 (1.04, 1.27)	0.01	1.20 (1.09, 1.33)	<0.001	1.14 (1.03, 1.26)	0.01
*CORONARY HEART DISEASE*										
<1/week	1139/ 34,231	594,854	1.00 reference		1.00 reference		1.00 reference		1.00 reference	
1/week to <1 day	365/ 12,048	212,095	1.04 (0.92, 1.17)	0.51	0.97 (0.85, 1.10)	0.65	1.00 (0.88, 1.14)	0.93	0.97 (0.85, 1.10)	0.65
≥ 1 serving/day	194/ 6475	115,749	1.20 (1.02, 1.40)	0.02	1.13 (0.95, 1.33)	0.16	1.17 (0.99, 1.37)	0.06	1.12 (0.95, 1.32)	0.17
***HEART FAILURE***										
<1/week	409/ 34,231	599,710	1.00 reference		1.00 reference		1.00 reference		1.00 reference	
1/week to <1 day	133/ 12,048	213,761	1.12 (0.92, 1.37)	0.26	0.90 (0.72, 1.12)	0.35	0.96 (0.71, 1.19)	0.71	0.90 (0.72, 1.12)	0.35
≥ 1 serving/day	67/ 6475	116,649	1.26 (0.97, 1.64)	0.09	0.97 (0.73, 1.28)	0.81	1.11 (0.84, 1.47)	0.45	0.96 (0.72, 1.28)	0.79
***STROKE***										
<1/week	1013/ 34,231	596,504	1.00 reference		1.00 reference		1.00 reference		1.00 reference	
1/week to <1 day	317/ 12,048	212,689	1.01 (0.89, 1.14)	0.92	0.98 (0.85, 1.12)	0.75	0.99 (0.87, 1.14)	0.93	0.98 (0.85, 1.13)	0.78
≥ 1 serving/day	184/ 6475	115,884	1.31 (1.11, 1.54)	<0.001	1.25 (1.05, 1.49)	0.01	1.28 (1.08, 1.52)	0.004	1.24 (1.04, 1.48)	0.01
***ISCHEMIC STROKE***										
<1/week	777/ 34,231	597,236	1.00 reference		1.00 reference		1.00 reference		1.00 reference	
1/week to <1 day	241/ 12,048	212,919	1.00 (0.86, 1.15)	0.97	0.98 (0.83, 1.14)	0.77	1.00 (0.86, 1.17)	0.96	0.98 (0.84, 1.15)	0.79
≥ 1 serving/day	136/ 6475	116,056	1.25 (1.04, 1.51)	0.02	1.18 (0.97, 1.45)	0.11	1.23 (1.00, 1.50)	0.05	1.18 (0.96, 1.44)	0.11
***HEMORRHAGIC STROKE***										
<1/week	150/ 34,231	602,073	1.00 reference		1.00 reference		1.00 reference		1.00 reference	
1/week to <1 day	53/ 12,048	214,496	1.08 (0.79, 1.49)	0.62	1.07 (0.76, 1.52)	0.69	1.02 (0.73, 1.45)	0.88	1.07 (0.76, 1.52)	0.69
≥ 1 serving/day	31/ 6475	116,922	1.37 (0.92, 2.02)	0.12	1.29 (0.83, 2.01)	0.25	1.27 (0.83, 1.95)	0.27	1.29 (0.83, 2.01)	0.25

Abbreviations: ASBs = artificially sweetened beverages; HR = hazard ratio; OS = observational study. Under/over energy reporters and those with baseline CVD, diabetes, and cancer were excluded from the analysis. Total CVD is a composite of incidence and death from CHD, stroke, heart failure, and coronary revascularization (CABG or PTCA). * Model 1 adjusted for age, region, smoking, and study arm. ** Model 2 adjusted for model 1+ ethnicity, education, marital status, BMI, physical activity, alcohol intake, energy intake, hypertension status, family history of CVD, family history of diabetes, hormone therapy use, and cholesterol-lowering medication use. *** Model 3 adjusted for model 2 + total protein intake, saturated fat intake, trans fat intake, and fiber, excluding without BMI. **** Model 4 adjusted for model 3 with BMI.

**Table 5:** Associations between added sugar, SSBs, ASBs, and the risk of cardiovascular outcomes in the random-effects network meta-analyses.

RR (95% CI) ^1^				
	Incident Cardiovascular Disease	Cardiovascular Mortality	Coronary Heart Disease	Stroke
ASB	1.21 (0.98–1.50) *n* = 2	1.26 (1.08–1.46) * n = 6	1.06 (0.95, 1.19) *n* = 4	1.19 (1.04, 1.36) * n = 5
SSB	1.14 (1.00–1.31) * n = 4	1.21 (1.07–1.36) * n = 10	1.17 (1.07–1.28) * n = 10	1.13 (1.03, 1.22) * n = 12
Added sugar	1.08 (0.86–1.36) *n* = 1	1.12 (0.96–1.32) *n* = 8	1.22 (1.04–1.42) * n = 2	1.10 (0.92, 1.33) *n* = 2
I-squared (*p* value)	71.3% * (<0.01)	62.4% * (<0.01)	38.2% (0.09)	43.6% * (0.03)

Abbreviations: ASB, artificially sweetened beverages; RR, relative risk; SSB, sugar-sweetened beverages. ^1^ The network meta-analysis was performed using frequentist methods with a random effects model. The study effect sizes for the highest versus the lowest category of exposure were calculated. * *p* < 0.05.

## Data Availability

The data presented in this study are available upon request from the corresponding author.

## Supplementary Materials

The following supporting information can be downloaded at: https://www.mdpi.com/article/10.3390/nu14204226/s1, Figure S1: Flow chart for the literature search, Figure S2: Plot of the added sugar, ASB, SSB, and CVD outcomes network; Table S1: Search terms and strategy for the systematic review and meta-analysis, Table S2: Subgroup analyses for added sugars and total SSBs and CVD outcomes according to baseline BMI category among 109,034 participants in the Women’s Health Initiative (CT + OS) (1993–2021), Table S3: Subgroup analyses for ASB servings and CVD outcomes according to baseline BMI category among 52,754 participants in the Women’s Health Initiative (OS) (1993–2021), Table S4: Subgroup analyses for added sugars and total SSBs and CVD outcomes according to baseline physical activity among 109,034 participants in the Women’s Health Initiative (CT + OS) (1993–2021), Table S5: Subgroup analyses for ASB servings and CVD outcomes according to baseline physical activity among 52,754 participants in the Women’s Health Initiative (OS) (1993–2021), Table S6: Sensitivity analyses for added sugars and total SSBs and total cardiovascular disease, coronary heart disease, and heart failure among participants in the Women’s Health Initiative (CT + OS) (1993–2021), Table S7: Sensitivity analyses for added sugars and total SSBs and total stroke and stroke subtypes among participants in the Women’s Health Initiative (CT + OS) (1993–2021), Table S8: Sensitivity analyses for ASBs with risk of cardiovascular disease outcomes among 52,754 participants in the Women’s Health Initiative (OS) (1993–2021), Table S9: Characteristics of included studies for the systematic review and meta-analysis, Table S10: Quality of included studies assessed by NOS scale and AHRQ standards, Table S11: Subgroup analyses for added sugar, SSB, ASB, and cardiovascular outcomes by sex category in the random effects network meta-analysis, Table S12: Sensitivity analyses for added sugar, SSB, ASB, and cardiovascular outcomes in the random effects network meta-analysis.
