# Supplementary material for: Added Sugar, Sugar-Sweetened Beverages, and Artificially Sweetened Beverages and Risk of Cardiovascular Disease: Findings from the Women’s Health Initiative and a Network Meta-Analysis of Prospective Studies
Source: Nutrients. 2022 Oct 11;14(20):4226. doi: 10.3390/nu14204226 (PMC9609206; doi:10.3390/nu14204226)
Supplement: Supplementary file 1 [file nutrients-14-04226-s001.zip › nutrients-1956824-supplementary.pdf]

Supplementary Materials

Table of Contents

Table S1 Search terms and strategy for the systematic review and meta-analysis 3

Table S2 Subgroup Analyses for Added Sugars and Total SSBs and CVD outcomes by baseline BMI category among 109,445 Participants in the Women's Health Initiative (CT+OS) (1993-2021) 4

Table S3 Subgroup Analyses for ASB servings and CVD outcomes by baseline BMI category among 52,754 Participants in the Women's Health Initiative (OS) (1993-2021) 7

Table S4 Subgroup Analyses for Added Sugars and Total SSBs and CVD outcomes by baseline physical activity among 109,445 Participants in the Women's Health Initiative (CT+OS) (1993-2021) 9

Table S5 Subgroup Analyses for ASB servings and CVD outcomes by baseline physical activity among 52,754 Participants in the Women's Health Initiative (OS) (1993-2021) 12

Table S6 Sensitivity Analyses for Added Sugars and Total SSBs and Total Cardiovascular Disease, Coronary Heart Disease and Heart Failure among Participants in the Women's Health Initiative (CT+OS) (1993-2021) 14

Table S7 Sensitivity Analyses for Added Sugars and Total SSBs and Total Stroke and Stroke Subtypes among Participants in the Women's Health Initiative (CT+OS) (1993-2021) 16

Table S8 Sensitivity analyses for ASBs with Risk of Cardiovascular Disease Outcomes among 52,754 Participants in the Women's Health Initiative (OS) (1993-2021) 18

Table S9 Characteristics of included studies for the systematic review and meta-analysis.................20

Table S10 Quality of included studies assessed by NOS scale and AHRQ standards.........................22

Table S11 Subgroup analyses for added Sugar, SSB, ASB, and cardiovascular outcomes by sex category in the random effects network meta-analysis...23

Table S12 Sensitivity analyses for added Sugar, SSB, ASB, and cardiovascular outcomes in the random effects network meta-analysis...24

Figure S1 Flow chart for the literature search...26

Figure S2 Plot of the added sugar, ASB, SSB, and CVD outcomes network.....................................27

Table S1. Search terms and strategy for the systematic review and meta-analysis

**Search terms:**<br>[added sugar or free sugar] OR [sugar-sweetened drink* or sugar sweetened beverage* or SSB or soft drink* or fruit drink* or soda] OR [artificially sweeten* or ASB or diet drink* or diet soft drink* or diet coke or diet coda or low calorie soda] AND [prospective or cohort* or longitudinal or follow-up] AND [inciden* or mortality or CVD or CHD or cardiovascular disease or cardiovascular mortality or cardiovascular death or cerebrovascular disease or coronary heart disease or coronary artery disease or stroke or ischemic stroke or haemorrhagic stroke or hemorrhagic stroke or myocardial infarction or ischemic heart diseases]
**Databases:**<br>Embase 1910 to Present,<br>Ovid Emcare 1995 to 2021 Week 5,<br>Ovid MEDLINE(R) and Epub Ahead of Print, In-Process & Other Non-Indexed Citations 1946 to February 09, 2021
**Inclusion criteria:**<br>1. All human participants;<br>2. Prospective cohort studies that examined the association between added sugar, SSB or ASB and cardiovascular outcomes;<br>3. The exposure interest was the dietary intake of added sugar, SSB or ASB;<br>4. Outcome: The outcomes were defined as coronary heart disease (defined as non-fatal myocardial infarction, angina, coronary revascularization (i.e., percutaneous transluminal coronary angioplasty or coronary artery bypass surgery, or coronary heart disease death), stroke (defined as fatal or nonfatal stroke), and composite cardiovascular disease (comprised of coronary heart disease and stroke). Outcomes were defined and diagnosed according to self-reported, medical record or clinical examination.<br>**Exclusion criteria:**<br>1. Intervention studies, review papers, comment, letters, news, notes, protocols, papers or abstracts from conference proceedings.<br>2. Articles without an abstract or full text in English.

Table S2. Subgroup Analyses for Added Sugars and Total SSBs and CVD outcomes by baseline BMI category among 109,034 Participants in the Women's Health Initiative (CT+OS) (1993-2021)

	BMI category			
**HR (95% CIs)**	<25 kg/m^2 (n=34,280)	25 to <30 kg/m^2 (n=32,394)	≥30kg/m^2 (n=25,093)	P for interaction
TOTAL CARDIOVASCULAR				
**Added sugars, % EAS**				0.29
<10%	1.00 [reference]	1.00 [reference]	1.00 [reference]	
10-14.9%	1.07 (0.98, 1.16)	0.98 (0.90, 1.06)	0.99 (0.92, 1.08)	
≥15.0%	1.18 (1.06, 1.32)	1.06 (0.95, 1.17)	1.05 (0.94, 1.17)	
**Total SSBs, servings**				0.65
<1/week	1.00 [reference]	1.00 [reference]	1.00 [reference]	
1/ week to <1 day	0.93 (0.86, 1.01)	0.96 (0.89, 1.03)	0.92 (0.86, 1.00)	
≥ 1 serving/day	1.19 (0.99, 1.42)	1.17 (1.00, 1.37)	1.06 (0.91, 1.23)	
CORONARY HEART DISEASE				
**Added sugars, % EAS**				0.01
<10%	1.00 [reference]	1.00 [reference]	1.00 [reference]	
10-14.9%	1.03 (0.90, 1.18)	0.94 (0.84, 1.07)	1.08 (0.95, 1.23)	
≥15.0%	1.39 (1.17, 1.65)	1.07 (0.91, 1.23)	1.22 (1.03, 1.44)	
**Total SSBs, servings**				0.63
<1/week	1.00 [reference]	1.00 [reference]	1.00 [reference]	
1/ week to <1 day	0.91 (0.80, 1.03)	0.92 (0.82, 1.03)	0.91 (0.81, 1.03)	
≥ 1 serving/day	1.27 (0.97, 1.67)	1.20 (0.94, 1.53)	1.09 (0.85, 1.38)	
HEART FAILURE				

**Added sugars, % EAS**				0.12
<10%	1.00 [reference]	1.00 [reference]	1.00 [reference]	
10-14.9%	1.01 (0.78, 1.30)	0.92 (0.73, 1.18)	0.95 (0.77, 1.17)	
≥15.0%	1.27 (0.91, 1.75)	0.82 (0.59, 1.16)	0.98 (0.74, 1.29)	
**Total SSBs, servings**				0.89
<1/week	1.00 [reference]	1.00 [reference]	1.00 [reference]	
1/ week to <1 day	1.06 (0.83, 1.35)	1.08 (0.85, 1.37)	0.96 (0.79, 1.18)	
≥ 1 serving/day	1.39 (0.82, 2.32)	1.23 (0.73, 2.06)	1.35 (0.93, 1.97)	
TOTAL STROKE				
**Added sugars, % EAS**				0.23
<10%	1.00 [reference]	1.00 [reference]	1.00 [reference]	
10-14.9%	1.04 (0.91, 1.19)	0.93 (0.82, 1.06)	0.97 (0.84, 1.11)	
≥15.0%	0.99 (0.81, 1.19)	1.08 (0.91, 1.28)	0.92 (0.77, 1.12)	
**Total SSBs, servings**				0.63
<1/week	1.00 [reference]	1.00 [reference]	1.00 [reference]	
1/ week to <1 day	0.92 (0.80, 1.05)	0.96 (0.84, 1.08)	0.91 (0.80, 1.05)	
≥ 1 serving/day	1.18 (0.88, 1.59)	1.13 (0.87, 1.46)	0.90 (0.69, 1.19)	
ISCHEMIC STROKE				
**Added sugars, % EAS**				0.60
<10%	1.00 [reference]	1.00 [reference]	1.00 [reference]	
10-14.9%	1.01 (0.86, 1.19)	0.86 (0.74, 1.00)	0.87 (0.74, 1.03)	
≥15.0%	0.93 (0.74, 1.16)	1.02 (0.84, 1.23)	0.90 (0.73, 1.12)	
**Total SSBs, servings**				0.62
<1/week	1.00 [reference]	1.00 [reference]	1.00 [reference]	

1/ week to <1 day	0.91 (0.78, 1.06)	0.99 (0.85, 1.14)	0.92 (0.79, 1.08)	
≥ 1 serving/day	1.18 (0.83, 1.67)	1.21 (0.90, 1.64)	0.89 (0.65, 1.22)	
HEMORRHAGIC STROKE				
**Added sugars, % EAS**				0.03
<10%	1.00 [reference]	1.00 [reference]	1.00 [reference]	
10-14.9%	1.04 (0.76, 1.42)	1.37 (0.96, 1.95)	1.45 (0.98, 2.15)	
≥15.0%	0.97 (0.62, 1.52)	1.64 (1.06, 2.54)	1.20 (0.70, 2.03)	
**Total SSBs, servings**				0.43
<1/week	1.00 [reference]	1.00 [reference]	1.00 [reference]	
1/ week to <1 day	0.82 (0.60, 1.11)	1.13 (0.81, 1.59)	0.96 (0.66, 1.39)	
≥ 1 serving/day	1.22 (0.62, 2.42)	1.20 (0.62, 2.36)	1.19 (0.59, 2.41)	

Abbreviations: CT=clinical trial; %EAS=percent energy from added sugar; HR, hazard ratio; OS=observational study; SSB=sugar sweetened beverages. Under/over energy reporters and those with baseline CVD, diabetes and cancer were excluded from the analysis.

*Model 1 adjusted for age, region, smoking, study arm, ethnicity, education, marital status, BMI, physical activity, alcohol intake, energy intake, hypertension status, family history of CVD, family history of diabetes, hormone therapy use, cholesterol-lowering medication use, total protein intake, saturated fat intake, trans fat intake, fiber intake

Table S3. Subgroup Analyses for ASB servings and CVD outcomes by baseline BMI category among 52,754 Participants in the Women's Health Initiative (OS) (1993-2021)

	BMI category			
**HR (95% CIs)**	<25 kg/m^2 (n=20,631)	25 to <30 kg/m^2 (n=15,888)	≥30kg/m^2 (n=9,742)	P for interaction
TOTAL CARDIOVASCULAR DISEASE				0.52
<1/week	1.00 [reference]	1.00 [reference]	1.00 [reference]	
1/ week to <1 day	0.98 (0.85, 1.14)	0.87 (0.77, 0.99)	1.09 (0.95, 1.26)	
≥ 1 serving/day	1.20 (0.98, 1.47)	1.09 (0.92, 1.28)	1.18 (1.00, 1.40)	
CORONARY HEART DISEASE				0.99
<1/week	1.00 [reference]	1.00 [reference]	1.00 [reference]	
1/ week to <1 day	0.98 (0.78, 1.23)	0.92 (0.75, 1.14)	1.02 (0.81, 1.29)	
≥ 1 serving/day	1.11 (0.79, 1.55)	1.14 (0.87, 1.48)	1.12 (0.85, 1.47)	
HEART FAILURE				0.54
<1/week	1.00 [reference]	1.00 [reference]	1.00 [reference]	
1/ week to <1 day	0.71 (0.45, 1.12)	0.90 (0.61, 1.32)	1.13 (0.81, 1.59)	
≥ 1 serving/day	0.92 (0.50, 1.72)	0.89 (0.51, 1.56)	1.18 (0.79, 1.76)	
TOTAL STROKE				0.06
<1/week	1.00 [reference]	1.00 [reference]	1.00 [reference]	
1/ week to <1 day	1.02 (0.80, 1.30)	0.84 (0.67, 1.05)	1.19 (0.91, 1.55)	
≥ 1 serving/day	1.46 (1.06, 2.03)	0.96 (0.72, 1.30)	1.43 (1.06, 1.92)	
ISCHEMIC STROKE				0.02
<1/week	1.00 [reference]	1.00 [reference]	1.00 [reference]	
1/ week to <1 day	0.98 (0.74, 1.30)	0.79 (0.62, 1.02)	1.36 (1.01, 1.83)	

≥ 1 serving/day	1.39 (0.95, 2.04)	0.84 (0.59, 1.19)	1.50 (1.07, 2.10)	
HEMORRHAGIC STROKE				0.68
<1/week	1.00 [reference]	1.00 [reference]	1.00 [reference]	
1/ week to <1 day	1.24 (0.73, 2.11)	1.06 (0.61, 1.84)	0.84 (0.37, 1.92)	
≥ 1 serving/day	1.41 (0.67, 3.00)	0.99 (0.46, 2.11)	1.49 (0.66, 3.35)	

Abbreviations: ASBs, artificially sweetened beverages; HR, hazard ratio; OS=observational study. Under/over energy reporters and those with baseline CVD, diabetes and cancer were excluded from the analysis. Total CVD is a composite of incidence and death of CHD, stroke, heart failure and coronary revascularization (CABG or PTCA).

*Model adjusted for age, region, smoking, ethnicity, education, marital status, BMI, physical activity, alcohol intake, energy intake, hypertension status, family history of CVD, family history of diabetes, hormone therapy use, cholesterol-lowering medication use, total protein intake, saturated fat intake, trans fat intake, fiber intake

Table S4. Subgroup Analyses for Added Sugars and Total SSBs and CVD outcomes by baseline physical activity among 109,034 Participants in the Women's Health Initiative (CT+OS) (1993-2021)

	**Physical activity category**		
**HR (95% CIs)**	≤median<br>(8.75 MET-hours/wk)<br>(n=51,635)	>median<br>(8.75 MET-hours/wk)<br>(n=57, 399)	P interaction
TOTAL CARDIOVASCULAR			
**Added sugars, % EAS**			0.68
<10%	1.00 [reference]	1.00 [reference]	
10-14.9%	1.01 (0.95, 1.08)	1.01 (0.94, 1.08)	
≥15.0%	1.09 (1.01, 1.19)	1.09 (0.99, 1.20)	
**Total SSBs, servings**			0.95
<1/week	1.00 [reference]	1.00 [reference]	
1/ week to <1 day	0.94 (0.87, 1.00)	0.93 (0.87, 0.99)	
≥ 1 serving/day	1.13 (0.98, 1.27)	1.14 (0.98, 1.32)	
CORONARY HEART DISEASE			
**Added sugars, % EAS**			0.21
<10%	1.00 [reference]	1.00 [reference]	
10-14.9%	1.04 (0.94, 1.15)	0.99 (0.89, 1.10)	
≥15.0%	1.27 (1.12, 1.44)	1.14 (0.99, 1.32)	
**Total SSBs, servings**			0.80
<1/week	1.00 [reference]	1.00 [reference]	
1/ week to <1 day	0.91 (0.83, 1.00)	0.90 (0.81, 1.00)	
≥ 1 serving/day	1.21 (1.00, 1.46)	1.13 (0.90, 1.43)	

HEART FAILURE			
**Added sugars, % EAS**			0.15
<10%	1.00 [reference]	1.00 [reference]	
10-14.9%	0.89 (0.75, 1.06)	1.05 (0.85, 1.29)	
≥15.0%	0.89 (0.71, 1.11)	1.22 (0.91, 1.62)	
**Total SSBs, servings**			0.15
<1/week	1.00 [reference]	1.00 [reference]	
1/ week to <1 day	0.98 (0.83, 1.15)	1.11 (0.90, 1.37)	
≥ 1 serving/day	1.16 (0.84, 1.62)	1.76 (1.14, 2.71)	
TOTAL STROKE			
**Added sugars, % EAS**			0.58
<10%	1.00 [reference]	1.00 [reference]	
10-14.9%	0.97 (0.87, 1.08)	0.99 (0.88, 1.11)	
≥15.0%	1.05 (0.91, 1.21)	0.95 (0.81, 1.11)	
**Total SSBs, servings**			0.86
<1/week	1.00 [reference]	1.00 [reference]	
1/ week to <1 day	0.95 (0.86, 1.05)	0.90 (0.81, 1.00)	
≥ 1 serving/day	1.10 (0.90, 1.36)	1.00 (0.78, 1.29)	
ISCHEMIC STROKE			
**Added sugars, % EAS**			0.52
<10%	1.00 [reference]	1.00 [reference]	
10-14.9%	0.92 (0.81, 1.05)	0.89 (0.78, 1.02)	
≥15.0%	1.02 (0.86, 1.20)	0.89 (0.74, 1.06)	
**Total SSBs, servings**			

<1/week	1.00 [reference]	1.00 [reference]	0.88
1/ week to <1 day	0.97 (0.86, 1.09)	0.90 (0.79, 1.02)	
≥ 1 serving/day	1.12 (0.88, 1.42)	1.04 (0.78, 1.38)	
HEMORRHAGIC STROKE			
**Added sugars, % EAS**			0.72
<10%	1.00 [reference]	1.00 [reference]	
10-14.9%	1.20 (0.91, 1.59)	1.28 (0.96, 1.70)	
≥15.0%	1.40 (0.98, 1.99)	1.09 (0.73, 1.64)	
**Total SSBs, servings**			0.86
<1/week	1.00 [reference]	1.00 [reference]	
1/ week to <1 day	1.01 (0.77, 1.32)	0.87 (0.66, 1.15)	
≥ 1 serving/day	1.32 (0.79, 2.21)	1.09 (0.60, 1.99)	

Abbreviations: CT=clinical trial; %EAS=percent energy from added sugar; HR, hazard ratio; OS=observational study; SSB=sugar sweetened beverages. Under/over energy reporters and those with baseline CVD, diabetes and cancer were excluded from the analysis.

*Model 1 adjusted for age, region, smoking, study arm, ethnicity, education, marital status, BMI, physical activity, alcohol intake, energy intake, hypertension status, family history of CVD, family history of diabetes, hormone therapy use, cholesterol-lowering medication use, total protein intake, saturated fat intake, trans fat intake, fiber intake

Table S5. Subgroup Analyses for ASB servings and CVD outcomes by baseline physical activity among 52,754 Participants in the Women's Health Initiative (OS) (1993-2021)

	**Physical activity category**		
**HR (95% CIs)**	≤median<br>(8.75 MET-hours/wk)<br>(n=20,058)	>median<br>(8.75 MET-hours/wk)<br>(n=26,234)	P for interaction
TOTAL CARDIOVASCULAR DISEASE			0.19
<1/week	1.00 [reference]	1.00 [reference]	
1/ week to <1 day	0.99 (0.87, 1.12)	0.95 (0.85, 1.06)	
≥ 1 serving/day	1.22 (1.06, 1.40)	1.07 (0.92, 1.24)	
CORONARY HEART DISEASE			0.74
<1/week	1.00 [reference]	1.00 [reference]	
1/ week to <1 day	1.07 (0.87, 1.29)	0.88 (0.74, 1.07)	
≥ 1 serving/day	1.16 (0.92, 1.47)	1.08 (0.85, 1.36)	
HEART FAILURE			0.83
<1/week	1.00 [reference]	1.00 [reference]	
1/ week to <1 day	0.89 (0.67, 1.20)	0.92 (0.66, 1.27)	
≥ 1 serving/day	0.99 (0.69, 1.43)	0.93 (0.59, 1.47)	
TOTAL STROKE			0.14
<1/week	1.00 [reference]	1.00 [reference]	
1/ week to <1 day	0.88 (0.72, 1.09)	1.08 (0.90, 1.30)	
≥ 1 serving/day	1.37 (1.08, 1.74)	1.12 (0.86, 1.45)	
ISCHEMIC STROKE			0.13
<1/week	1.00 [reference]	1.00 [reference]	

1/ week to <1 day	0.94 (0.74, 1.19)	1.02 (0.83, 1.26)	
≥ 1 serving/day	1.31 (0.99, 1.73)	1.04 (0.77, 1.40)	
HEMORRHAGIC STROKE			0.96
<1/week	1.00 [reference]	1.00 [reference]	
1/ week to <1 day	0.86 (0.51, 1.45)	1.34 (0.84, 2.14)	
≥ 1 serving/day	1.32 (0.73, 2.40)	1.29 (0.68, 2.49)	

Abbreviations: ASBs, artificially sweetened beverages; HR, hazard ratio; OS=observational study. Under/over energy reporters and those with baseline CVD, diabetes and cancer were excluded from the analysis. Total CVD is a composite of incidence and death of CHD, stroke, heart failure and coronary revascularization (CABG or PTCA).

*Model adjusted for age, region, smoking, ethnicity, education, marital status, BMI, physical activity, alcohol intake, energy intake, hypertension status, family history of CVD, family history of diabetes, hormone therapy use, cholesterol-lowering medication use, total protein intake, saturated fat intake, trans fat intake, fiber intake

Table S6. Sensitivity Analyses for Added Sugars and Total SSBs and Total Cardiovascular Disease, Coronary Heart Disease and Heart Failure among Participants in the Women's Health Initiative (CT+OS) (1993-2021)

	1. OS participants only (n=52,754)		*2. Removing diagnosis in first 3 years*	
	HR (95% CI)	*P* value	HR (95% CI)	*P* value
TOTAL CARDIOVASCULAR DISEASE				
**%EAS**				
<10%	1.00 [reference]		1.00 [reference]	
10-14.9%	1.01 (0.95, 1.08)	0.69	1.02 (0.97, 1.08	0.39
≥15.0%	1.11 (1.01, 1.21)	0.03	1.05 (0.98, 1.12)	0.16
**Total SSBs**				
<1/week	1.00 [reference]		1.00 [reference]	
1/ week to <1 day	1.00 (0.93, 1.07)	0.98	1.01 (0.96, 1.06)	0.66
≥ 1 serving/day	1.34 (1.19, 1.52)	<0.001	1.24 (1.11, 1.37)	<0.001
CORONARY HEART DISEASE				
**%EAS**				
<10%	1.00 [reference]		1.00 [reference]	
10-14.9%	1.02 (0.91, 1.14)	0.71	1.03 (0.96, 1.12)	0.39
≥15.0%	1.18 (1.02, 1.36)	0.02	1.17 (1.05, 1.30)	0.002
**Total SSBs**				
<1/week	1.00 [reference]		1.00 [reference]	
1/ week to <1 day	0.99 (0.88, 1.11)	0.88	0.98 (0.91, 1.06)	0.61
≥ 1 serving/day	1.40 (1.15, 1.71)	0.001	1.35 (1.15, 1.58)	<0.001

HEART FAILURE				
**%EAS**				
<10%	1.00 [reference]		1.00 [reference]	
10-14.9%	0.91 (0.76, 1.10)	0.35	0.96 (0.82, 1.12)	0.64
≥15.0%	1.08 (0.86, 1.38)	0.48	0.88 (0.71, 1.08)	0.24
**Total SSBs**				
<1/week	1.00 [reference]		1.00 [reference]	
1/ week to <1 day	1.10 (0.90, 1.34)	0.35	1.09 (0.93, 1.27)	0.70
≥ 1 serving/day	1.58 (1.13, 2.20)	0.006	1.18 (0.85 1.62)	0.32

Abbreviations: CHD=coronary heart disease; CT=clinical trial; CVD, cardiovascular disease; %EAS=percent energy from added sugar; HF=heart failure; HR, hazard ratio; OS=observational study; SSB=sugar sweetened beverages. Under/over energy reporters and those with baseline CVD, diabetes and cancer were excluded from the analysis. Total CVD is a composite of incidence and death of CHD, stroke, heart failure and coronary revascularization (CABG or PTCA).

Results are for model 3, adjusted for age, region, smoking, study arm (except sensitivity analysis 1), ethnicity, education, marital status, BMI, physical activity, alcohol intake, energy intake, hypertension status, family history of CVD, family history of diabetes, hormone therapy use, cholesterol-lowering medication use, total protein intake, saturated fat intake, trans fat intake, and fiber intake.

Table S7. Sensitivity Analyses for Added Sugars and Total SSBs and Total Stroke and Stroke Subtypes among Participants in the Women's Health Initiative (CT+OS) (1993-2021)

	1. OS participants only (n=52,754)		***2. Removing diagnosis in first 3 years***	
	HR (95% CI)	*P* value	HR (95% CI)	*P* value
TOTAL STROKE				
**%EAS**				
<10%	1.00 [reference]		1.00 [reference]	
10-14.9%	0.98 (0.87, 1.10)	0.73	0.97 (0.89, 1.06)	0.51
≥15.0%	1.03 (0.89, 1.21)	0.64	0.97 (0.87, 1.09)	0.61
**Total SSBs**				
<1/week	1.00 [reference]		1.00 [reference]	
1/ week to <1 day	0.97 (0.86, 1.10)	0.66	1.03 (0.95, 1.12)	0.43
≥ 1 serving/day	1.32 (1.07, 1.63)	0.009	1.27 (1.06, 1.51)	0.008
ISCHEMIC STROKE				
**%EAS**				
<10%	1.00 [reference]		1.00 [reference]	
10-14.9%	0.92 (0.81, 1.06)	0.26	0.91 (0.83, 1.01)	0.07
≥15.0%	0.93 (0.77, 1.11)	0.42	0.94 (0.82, 1.07)	0.37
**Total SSBs**				
<1/week	1.00 [reference]		1.00 [reference]	
1/ week to <1 day	0.98 (0.85, 1.14)	0.82	1.00 (0.82, 1.22)	0.98
≥ 1 serving/day	1.32 (1.03, 1.68)	0.02	1.37 (0.92, 2.05)	0.12

HEMORRHAGIC STROKE				
**%EAS**				
<10%	1.00 [reference]		1.00 [reference]	
10-14.9%	1.27 (0.95, 1.70)	0.11	1.20 (0.96, 1.49)	0.10
≥15.0%	1.54 (1.06, 2.22)	0.02	1.11 (0.82, 1.49)	0.50
**Total SSBs**				
<1/week	1.00 [reference]		1.00 [reference]	
1/ week to <1 day	0.99 (0.73, 1.34)	0.94	1.00 (0.81, 1.24)	0.99
≥ 1 serving/day	1.57 (0.94, 2.60)	0.08	1.58 (1.03, 2.43)	0.04

Abbreviations: CHD=coronary heart disease; CT=clinical trial; CVD, cardiovascular disease; %EAS=percent energy from added sugar; HF=heart failure; HR, hazard ratio; OS=observational study; SSB=sugar sweetened beverages. Under/over energy reporters and those with baseline CVD, diabetes and cancer were excluded from the analysis. Total CVD is a composite of incidence and death of CHD, stroke, heart failure and coronary revascularization (CABG or PTCA).

Results are for model 3, adjusted for age, region, smoking, study arm (except sensitivity analysis 1), ethnicity, education, marital status, BMI, physical activity, alcohol intake, energy intake, hypertension status, family history of CVD, family history of diabetes, hormone therapy use, cholesterol-lowering medication use, total protein intake, saturated fat intake, trans fat intake, and fiber intake.

Table S8. Sensitivity analyses for ASBs with Risk of Cardiovascular Disease Outcomes among 52,754 Participants in the Women's Health Initiative (OS) (1993-2021)

	**Removing diagnoses in first 3 years**	
CARDIOVASCULAR DISEASE	HR (95% CI)	*P* value
<1/week	1.00 [reference]	
1/ week to <1 day	0.96 (0.88, 1.05)	0.39
≥ 1 serving/day	1.10 (0.98, 1.23)	0.08
CORONARY HEART DISEASE		
<1/week	1.00 [reference]	
1/ week to <1 day	0.97 (0.85, 1.11)	0.67
≥ 1 serving/day	1.10 (0.93, 1.31)	0.27
HEART FAILURE		
<1/week	1.00 [reference]	
1/ week to <1 day	0.92 (0.72, 1.17)	0.49
≥ 1 serving/day	0.92 (0.66, 1.29)	0.63
TOTAL STROKE		
<1/week	1.00 [reference]	
1/ week to <1 day	0.97 (0.84, 1.12)	0.67
≥ 1 serving/day	1.14 (0.95, 1.38)	0.17
ISCHEMIC STROKE		
<1/week	1.00 [reference]	
1/ week to <1 day	1.00 (0.85, 1.18)	0.99
≥ 1 serving/day	1.07 (0.86, 1.33)	0.55

HEMORRHAGIC STROKE		
<1/week	1.00 [reference]	
1/ week to <1 day	0.90 (0.61, 1.31)	0.58
≥ 1 serving/day	1.14 (0.71, 1.83)	0.60

Abbreviations: ASBs, artificially sweetened beverages; HR, hazard ratio; OS=observational study. Under/over energy reporters and those with baseline CVD, diabetes and cancer were excluded from the analysis. Total CVD is a composite of incidence and death of CHD, stroke, heart failure and coronary revascularization (CABG or PTCA). Models are adjusted for age, region, smoking, ethnicity, education, marital status, BMI, physical activity, alcohol intake, energy intake, hypertension status, family history of CVD, family history of diabetes, hormone therapy use, cholesterol-lowering medication use, total protein intake, saturated fat intake, trans fat intake, fiber intake.

Table S9. Characteristics of included studies for the systematic review and meta-analysis

Author, year	Country	Cohort name	Sex	Age at entry	Sample size	Outcome	Exposure	Follow up years
Fung, 2009	USA	NHS	Women	34-59	88,520	CHD	SSB, ASB	24
Bernstein, 2012	United States (USA)	Health Professionals Follow-Up Study (HPFS)+ Nurses' Health Study (NHS)	Both	34-59 (NHS), 40-75 (HPFS)	127,456	Stroke	SSB, ASB	22 (HPFS), 28 (NHS)
de Koning, 2012	USA	HPFS	Men	40-75	42,883	Coronary heart disease (CHD)	SSB, ASB	22
Eshak, 2012	Japan	Japan Public Health Centre–based prospective	Both	40-59	39,786	CHD, Stroke	SSB	18
Gardener, 2012	USA	Northern Manhattan Study	Men	~69	2,564	CVD incidence, CVD death, myocardial infraction (MI), Stroke	SSB, ASB	10
Larsson, 2014	Sweden	Cohort of Swedish Men + Swedish Mammography Cohort	Both	45-79 (Cohort of Swedish Men) + 49-83 (Swedish Mammography Cohort)	68,459	Stroke	SSB	10.3
Tasevska, 2014	USA	NIH-AARP Diet and Health Study	Both	50-71	353,751	CVD mortality	Added Sugar	up to 13
Yang, 2014	USA	National Health and Nutrition Examination Survey III (1988-1994)	Both	≥20	11,733	CVD mortality	Added Sugar	14.6
Sonested, 2015	Sweden	Malmö Diet and Cancer Study cohort	Both	44-74	26,445	CVD incidence, CHD, Stroke	SSB	14
Odegaard, 2015	Singapore	Singapore Chinese Health Study	Both	45–74	52,584	CVD mortality	SSB	16.3
Barrington, 2016	USA	Vitamins and Lifestyle study	Both	50-76	69,582	CVD mortality	SSB	6.9

Pase, 2017	USA	Framingham Heart Study	Both	≥45	2,888	Stroke	SSB, ASB	10
Liu, 2018	Hong Kong	Mr and Ms Osteoporosis of Hong Kong	Both	≥65	3,416	CVD mortality	Added Sugar	11.1
Collin, 2019	USA	Geographic and Racial Differences in Stroke study	Both	≥45	13,440	CHD	SSB	6
Malik 2019	USA	NHS+HPFS	Both	30–55(NHS)+40–75(HPFS)	116,383	CVD mortality	SSB, ASB	34
Mullee, 2019	Ten European countries	European Prospective Investigation into Cancer and Nutrition	Both	50.8	451,743	CVD mortality	SSB, ASB	16.4
Ramne, 2019	Swedish	Malmö Diet and Cancer Study	Both	44–73	24,272	CVD mortality	Added Sugar	~20
Janzi, 2020	Sweden	Malmö Diet and Cancer Study cohort	Both	44-74	25,877	Stroke, CHD	Added sugar, SSB	19.5
Pacheco, 2020	USA	California Teachers Study	Women	52.1±13.4	106,178	CVD incidence, MI, Stroke	SSB	20
Zhang, 2020	USA	National Health and Nutrition Examination Survey 1999-2014	Both	≥20	31,402	CVD mortality	Added Sugar, SSB, ASB	7.9
Huang, 2021	Japan	Japan Public Health Centre–based prospective	Both	45-74	70486 (7,118 men, 4,693 women)	CVD incidence, stroke	SSB	17.1

Table S10. Quality of included studies assessed by NOS scale and AHRQ standards

Author, year	Selection	Comparability	Outcome	Total NOS Score	AHRQ Standards
Fung, 2009	3	2	2	7	good
Bernstein, 2012	2	1	3	6	fair
de Koning, 2012	3	2	3	8	good
Eshak, 2012	3	2	3	8	good
Gardener, 2012	4	2	3	9	good
Larsson, 2014	3	2	2	7	good
Tasevska, 2014	3	2	2	7	good
Yang, 2014	4	2	2	8	good
Sonested, 2015	3	2	2	7	good
Odegaard, 2015	4	2	3	9	good
Barrington, 2016	3	2	3	8	good
Pase, 2017	3	2	2	7	good
Liu, 2018	3	2	3	8	good
Collin, 2019	3	2	3	8	good
Malik 2019	3	2	3	8	good
Mullee, 2019	4	2	3	9	good
Ramne, 2019	3	2	2	7	good
Janzi, 2020	4	2	3	9	good
Pacheco, 2020	3	2	2	7	good
Zhang, 2020	4	2	2	8	good
Huang, 2021	4	2	3	9	good

Abbreviations: AHRQ, the Agency for Healthcare Research and Quality; NOS, Newcastle-Ottawa Scale.

Table S11. Subgroup analyses for added Sugar, SSB, ASB, and cardiovascular outcomes by sex category in the random effects network meta-analysis

	Sex category		
**RR (95% CIs)**	Men only (n=24)		Women only (n=28)
INCIDENT CARDIOVASCULAR DISEASE			
**Lowest exposure**	1.00 [reference]	**Lowest exposure**	1.00 [reference]
**Added sugar (n=0)**	NA	**Added sugar (n=1)**	1.08 (1.00, 1.16) *
**SSB (n=1)**	1.09 (0.82, 1.45)	**SSB (n=2)**	1.25 (1.15, 1.35) *
**ASB (n=1)**	1.44 (1.02, 2.03) *	**ASB (n=1)**	1.14 (1.03, 1.27) *
CARDIOVASCULAR MORTALITY			
**Lowest exposure**	1.00 [reference]	**Lowest exposure**	1.00 [reference]
**Added sugar (n=3)**	0.92 (0.73, 1.15)	**Added sugar (n=3)**	1.17 (0.80, 1.71)
**SSB (n=4)**	1.17 (0.99, 1.38)	**SSB (n=3)**	1.34 (1.01, 1.77) *
**ASB (n=3)**	1.25 (1.03, 1.51) *	**ASB (n=2)**	1.30 (0.94, 1.80)
CORONARY HEART DISEASE			
**Lowest exposure**	1.00 [reference]	**Lowest exposure**	1.00 [reference]
**Added sugar (n=0)**	NA	**Added sugar (n=1)**	1.20 (1.09, 1.32) *
**SSB (n=3)**	1.13 (0.92, 1.38)	**SSB (n=4)**	1.30 (1.17, 1.45) *
**ASB (n=2)**	1.07 (0.85, 1.34)	**ASB (n=2)**	1.13 (1.01, 1.28) *
STROKE			
**Lowest exposure**	1.00 [reference]	**Lowest exposure**	1.00 [reference]
**Added sugar (n=0)**	NA	**Added sugar (n=1)**	0.99 (0.89, 1.10)
**SSB (n=4)**	1.02 (0.83, 1.25)	**SSB (n=5)**	1.22 (1.12, 1.32) *
**ASB (n=2)**	1.13 (0.83, 1.54)	**ASB (n=2)**	1.19 (1.08, 1.31) *

Abbreviations: ASB, artificial sweetened beverages; RR, relative risk; SSB, sugar sweetened beverage

Table S12. Sensitivity analyses for added Sugar, SSB, ASB, and cardiovascular outcomes in the random effects network meta-analysis

	RR (95% CIs)		
INCIDENT CARDIOVASCULAR DISEASE			
	1. Excluding the present study (SSB)	2. Excluding 1 & Sonestedt 2015 (SSB)	3. Excluding 2 & the present study (added sugar)
**Lowest exposure**	1.00 [reference]	1.00 [reference]	1.00 [reference]
**Added sugar**	1.08 (0.89, 1.31)	1.08 (1.01, 1.15)	NA
**SSB**	1.09 (0.95, 1.25)	1.18 (1.05, 1.31)	1.18 (1.05, 1.31)
**ASB**	1.20 (1.00, 1.44)	1.16 (1.06, 1.28)	1.16 (1.06, 1.28)
**I-squared**	53.5% (0.0, 84.6)	0.0% (0.0, 89.6)	0.0% (0.0, 89.6)
CARDIOVASCULAR MORTALITY			
	1. Excluding Tasevska 2014.men (added sugar)	2. Excluding 1 & Tasevska 2014.women (added sugar)	3. Excluding 2 & Mullee 2019.men (ASB)
**Lowest exposure**	1.00 [reference]	1.00 [reference]	1.00 [reference]
**Added sugar**	1.23 (1.01, 1.50)	1.45 (1.15, 1.82)	1.45 (1.16, 1.81)
**SSB**	1.21 (1.07, 1.37)	1.21 (1.09, 1.34)	1.21 (1.09, 1.33)
**ASB**	1.26 (1.07, 1.47)	1.25 (1.09, 1.43)	1.19 (1.04, 1.38)
**I-squared**	59.0% (33.7, 74.7)	45.7% (8.3, 67.9)	40.1% (0.0, 65.3)
CORONARY HEART DISEASE			
	1. Excluding de Koning 2012 (ASB)	2. Excluding 1 & the present study (SSB)	3. Excluding 2 & Sonestedt 2015 (SSB)
**Lowest exposure**	1.00 [reference]	1.00 [reference]	1.00 [reference]
**Added sugar**	1.21 (1.05, 1.40)	1.21 (1.10, 1.33)	1.21 (1.10, 1.32)
**SSB**	1.17 (1.07, 1.27)	1.13 (1.05, 1.21)	1.17 (1.08, 1.27)
**ASB**	1.13 (0.98, 1.32)	1.13 (1.00, 1.28)	1.13 (1.01, 1.28)
**I-squared**	29.4% (0.0, 66.2)	2.7% (0.0, 65.7)	0.0% (0.0, 67.6)
STROKE			
	1. Excluding the present study (added sugar)	2. Excluding 1 & Eshak 2012.men (SSB)	3. Excluding 2 & Sonestedt 2015 (SSB)
**Lowest exposure**	1.00 [reference]	1.00 [reference]	1.00 [reference]

**Added sugar**	1.57 (1.09, 2.26)	1.57 (1.12, 2.19)	1.57 (1.12, 2.19)
**SSB**	1.13 (1.05, 1.22)	1.16 (1.09, 1.24)	1.20 (1.12, 1.28)
**ASB**	1.18 (1.05, 1.33)	1.18 (1.08, 1.28)	1.18 (1.08, 1.28)
**I-squared**	31.0% (0.0, 62.2)	0.0% (0.0, 53.6)	0.0% (0.0, 55.0)

Abbreviations: ASB, artificial sweetened beverages; RR, relative risk; SSB, sugar sweetened beverages.

Top three studies causing most significant changes to I-squared in the leave-one-out meta-analysis were excluded stepwise in the sensitivity analysis

Figure S1. Flow chart for the literature search

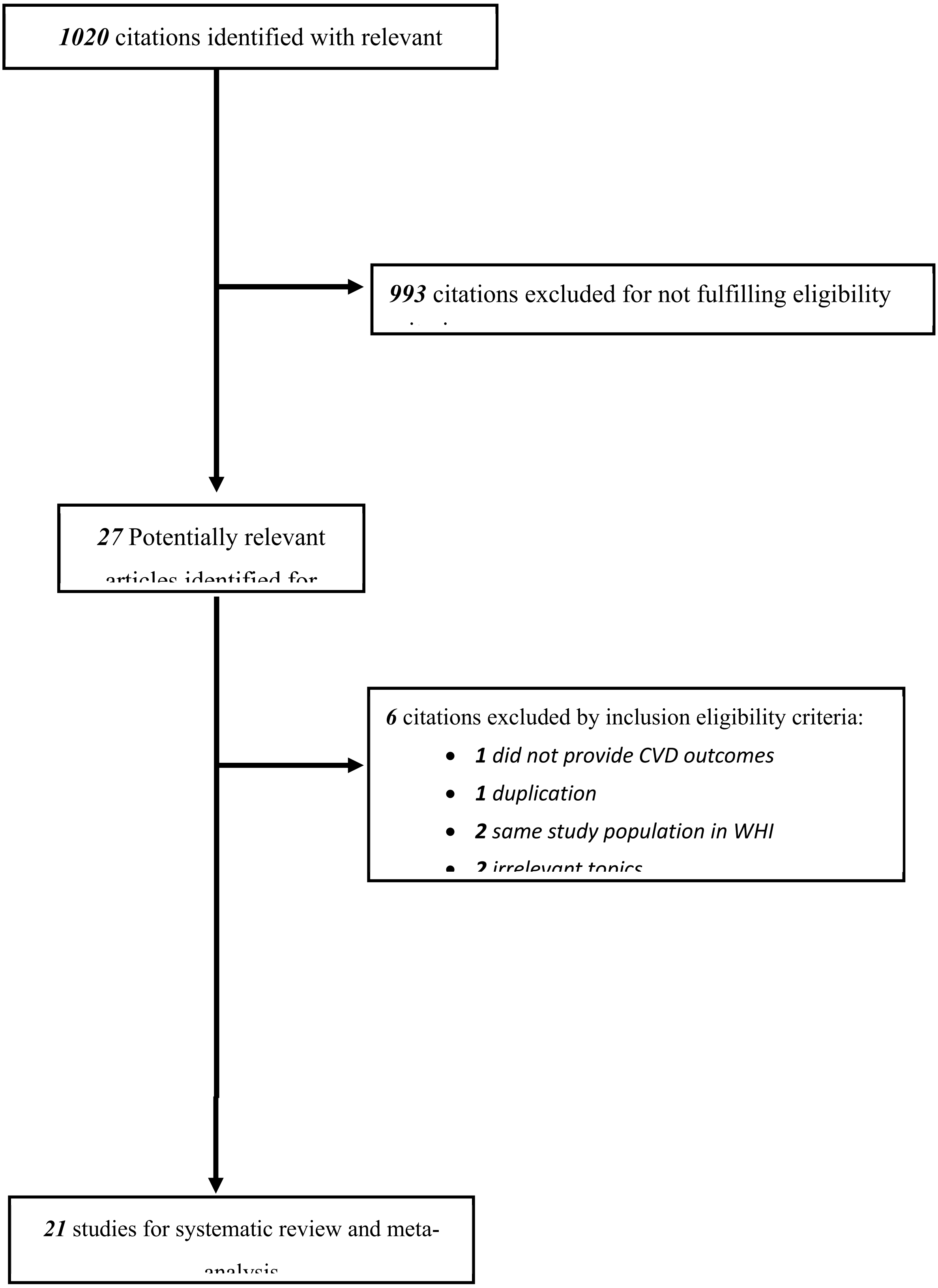

Figure S2. Plot of the added sugar, ASB, SSB, and CVD outcomes network

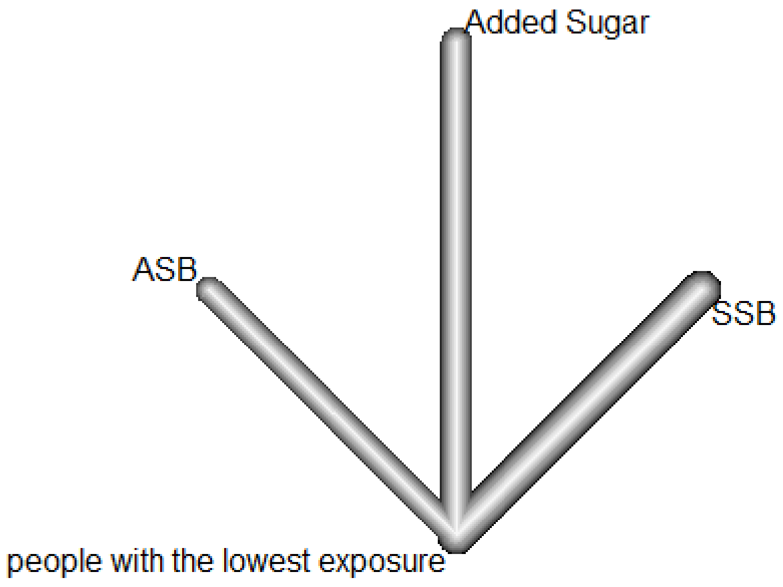

The network graph was generated based on the random effects network meta-analysis on added sugar, ASB, SSB, and CVD outcomes. Thicker lines indicating more publications included in the analysis (Added sugar, 13 records; ASB, 17 records; SSB, 38 records).

Abbreviations: ASB: artificially sweetened beverages; SSB: sugar-sweetened beverages
